# A Balanced Memory Network

**DOI:** 10.1371/journal.pcbi.0030141

**Published:** 2007-09-07

**Authors:** Yasser Roudi, Peter E Latham

**Affiliations:** Gatsby Computational Neuroscience Unit, University College London, London, United Kingdom; University College London, United Kingdom

## Abstract

A fundamental problem in neuroscience is understanding how working memory—the ability to store information at intermediate timescales, like tens of seconds—is implemented in realistic neuronal networks. The most likely candidate mechanism is the attractor network, and a great deal of effort has gone toward investigating it theoretically. Yet, despite almost a quarter century of intense work, attractor networks are not fully understood. In particular, there are still two unanswered questions. First, how is it that attractor networks exhibit irregular firing, as is observed experimentally during working memory tasks? And second, how many memories can be stored under biologically realistic conditions? Here we answer both questions by studying an attractor neural network in which inhibition and excitation balance each other. Using mean-field analysis, we derive a three-variable description of attractor networks. From this description it follows that irregular firing can exist only if the number of neurons involved in a memory is large. The same mean-field analysis also shows that the number of memories that can be stored in a network scales with the number of excitatory connections, a result that has been suggested for simple models but never shown for realistic ones. Both of these predictions are verified using simulations with large networks of spiking neurons.

## Author Summary

A critical component of cognition is memory—the ability to store information, and to readily retrieve it on cue. Existing models postulate that recalled items are represented by self-sustained activity; that is, they are represented by activity that can exist in the absence of input. These models, however, are incomplete, in the sense that they do not explain two salient experimentally observed features of persistent activity: low firing rates and high neuronal variability. Here we propose a model that can explain both. The model makes two predictions: changes in synaptic weights during learning should be much smaller than the background weights, and the fraction of neurons selective for a memory should be above some threshold. Experimental confirmation of these predictions would provide strong support for the model, and constitute an important step toward a complete theory of memory storage and retrieval.

## Introduction

A critical component of any cognitive system is working memory—a mechanism for storing information about past events, and for accessing that information at later times. Without such a mechanism, even simple tasks, such as deciding whether to wear a heavy jacket or a light sweater after hearing the weather report, would be impossible. Although it is not known exactly how storage and retrieval of information is implemented in neural systems, a very natural way is through attractor networks. In such networks, transient events in the world trigger stable patterns of activity in the brain, so by looking at the pattern of activity at the current time, other areas in the brain can know something about what happened in the past.

There is now considerable experimental evidence for attractor networks in areas such as inferior temporal cortex [1–3], prefrontal cortex [4–9], and hippocampus [10,11]. And from a theoretical standpoint, it is well understood how attractor networks could be implemented in neuronal networks, at least in principle. Essentially, all that is needed is an increase in the connection strength among subpopulations of neurons. If the increase is sufficiently large, then each subpopulation can be active without input, and thus “remember” events that happened in the past.

While the basic theory of attractor networks has been known for some time [12–14], moving past the “in principle” qualifier, and understanding how attractors could be implemented in realistic, spiking networks, has been difficult. This is because the original Hopfield model violated several important principles: neurons did not obey Dale's law; when a memory was activated, neurons fired near saturation, much higher than is observed experimentally in working memory tasks [1,15]; and there was no null background state—no state in which all neurons fired at low rates.

Most of these problems have been solved. The first, that Dale's law was violated, was solved by “clipping” synaptic weights; that is, by using the Hopfield prescription [12], assigning neurons to be either excitatory or inhibitory, and then setting any weights of the wrong sign to zero [16,17]. The second, building a Hopfield-type network with low firing rate, was solved by adding appropriate inhibition [18–23] (importantly, this was a nontrivial fix; for discussion, see [23]). The third problem, no null background, was solved either by making the units sufficiently stochastic [18–21] or adding external input [14,20–23].

In spite of these advancements, there are still two fundamental open questions. One is: how can we understand the highly irregular firing that is observed experimentally in working memory tasks [24]? Answering this question is important because irregular firing is thought to play a critical role both in how fast computations are carried out [25] and in the ability of networks to perform statistical inference [26]. Answering it is hard, though, because, as pointed out in [27], with naive scaling the net synaptic drive to the foreground neurons (the neurons that fire at elevated rate during memory) is proportional to the number of connections per neuron. Consequently, because of the high connectivity observed in cortex, the mean synaptic drive is much larger than the fluctuations, which implies that the foreground neurons should fire regularly. Moreover, as pointed out by Renart et al. [28], even for models that move beyond the naive scaling and produce irregularly firing neurons, the foreground neurons still tend to fire more regularly than the background neurons, something that is inconsistent with experiments [24].

Several studies have attempted to get around this problem, either directly or indirectly [22,27–29]. Most of them, however, did not investigate the scaling of the network parameters with its size (i.e., with the number of neurons and connections). So, although parameters were found which led to irregular activity, it was not clear how those parameters should scale as the size of the network increased to realistic values. In the two that did investigate scaling [27,28], irregular firing was possible only if a small fraction of neurons was involved in each memory; i.e., only if the coding level was very small. Although there have been no direct measurements of the coding level during persistent activity, at least to our knowledge, experiments in superior temporal sulcus [30] suggest that it is much larger than the one used in these models. We should point out, though, that the model of Renart et al. [28] is the only one in which the foreground neurons are at least as regular as the background neurons.

The second open question is: what is the storage capacity of realistic attractor networks? That is, how many different memories can be stored in a single network? Answering this is critical for understanding the highly flexible and seemingly unbounded memory capacity observed in animals. For simple, albeit unrealistic, models the answer is known: as shown in the seminal work of Amit, Gutfreund, and Sompolinsky [31], the number of memories that can be stored in a classical Hopfield network [12] is about 0.14 times the number of neurons. For slightly more realistic networks the answer is also known [16,19,21,27,32–38]. However, even these more realistic studies lacked biological plausibility in at least one way: connectivity was all–all rather than sparse [19,21,33,38], the neurons were binary (either on or off, with nothing in between) [16,19,21,32,33,37], there was no null background [16,32,33,35,37,38], the firing rate in the foreground state was higher than is observed experimentally [16,27,32,33,36,37], or the coding level was very small [27,36].

Here we answer both questions: we show, for realistic networks of spiking neurons, how irregular firing can be achieved, and we compute the storage capacity. Our analysis uses relatively standard mean-field techniques, and requires only one assumption: neurons in the network fire asynchronously. Given this assumption, we first show that neurons fire irregularly only if the coding level is above some threshold, although a feature of our model is that the foreground neurons are slightly more regular than the background neurons. We then show that the maximum number of memories in our network—the capacity—is proportional to the number of connections per neuron, a result that is consistent with the simplified models discussed above. These predictions are verified with simulations of biologically plausible networks of spiking neurons.

## Results

### Model

To address analytically the issues of irregularity and storage capacity in attractor networks, we consider a model in which neurons are described by their firing rates. Although firing rate models typically provide a fairly accurate description of network behaviour when the neurons are firing asynchronously [39,40], they do not capture all features of realistic networks. Therefore, we verify all of our predictions with large-scale simulations of spiking neurons.

Our network consists of two populations, one excitatory and one inhibitory, with *N_E_* neurons in the former and *N*_I_ in the latter. (In general we use *E* for excitation and *I* for inhibition.) We represent the firing rate of the *i*th neuron in pool *Q*(=*E*,*I*) by *v_Qi_*. As we show in the section “Fast fluctuations,” and discuss below, the time evolution equations for the firing rates are given by

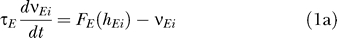


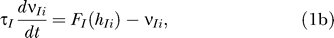
where *τ_E_* and *τ_I_* are the excitatory and inhibitory time constants, *h_Qi_* is the synaptic input to the *i*th neuron in pool *Q*, and *F_Q_*(*h*) is a function that tells us the steady state firing rate of a neuron receiving synaptic input *h*. This function, which has a relatively stereotyped quasi-sigmoidal shape, can be determined analytically (or semi-analytically) for specific noise models [41–43], and numerically for more realistic models [40]. The synaptic drive, *h_Qi_*, is related to the activity of the presynaptic neurons via

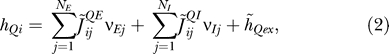
where 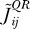 is the synaptic weight from the *j*th neuron in pool *R* to the *i*th neuron in pool *Q*, and 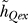 is the external, purely excitatory, input to neurons in pool *Q*. Finally, the steady-state firing rate of each neuron is determined by setting *dv_Ei_*/*dt* and *dv_Ii_*/*dt* to zero, yielding the equation

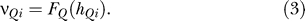



The bulk of our analysis focuses on solving Equation 3; we use the dynamics, Equation 1, only when investigating stability. Our goal is to determine the conditions that support retrieval states—states such that subpopulations of neurons have elevated firing rates.

Since the gain functions, *F_Q_*(*h*), that we use in Equation 1 play such a central role in our analysis, we briefly justify them here; for additional details, see the section “Fast fluctuations.” These gain functions come from an average over the fast temporal fluctuations of the synaptic input—basically, filtered spikes. Calculating the temporal fluctuations self-consistently is a hard problem [44], but, fortunately, it's not a problem we have to solve. As we show in the section “Fast fluctuations,” in the limit that each neuron receives a large number of connections, the temporal fluctuations experienced by all the excitatory neurons have the same statistics, as do the temporal fluctuations experienced by all the inhibitory neurons. Thus, we can use a single function, *F_E_*(*h*), for the excitatory neurons, and another function, *F_I_*(*h*), for the inhibitory ones. Of course, we won't be able to calculate the shape of *F_Q_* without knowing the structure of the temporal fluctuations. However, as we show below, the precise shapes of the gain functions don't play a strong role in our analysis.

#### Connectivity.

The main determinant of network behaviour, at least in this model, is the set of connection strengths, the 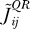 . To choose connection strengths that will lead to attractors, we build on the model proposed by Hopfield more than two decades ago [12]. In that model, random patterns are stored via a Hebbian learning rule, so connection strengths among neurons have the form

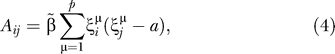
where *A_ij_* is the strength of the connection from neuron *j* to neuron *i*, 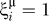 if neuron *i* participates in pattern *μ* and 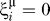 otherwise, 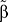 is a constant that determines the memory strength, and *p* is the number of patterns. For each neuron, the probability of participating in a given pattern, *μ*, is equal to the coding level, which we denote *a*. Thus,

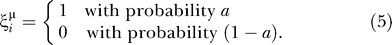



With this definition, the term 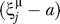 in Equation 4 ensures that, on average, ∑*_j_A_ij_* is zero. Thus, the learning rule does not change the total synaptic weight onto a neuron, a form of postsynaptic normalisation that has been observed experimentally in cultured networks [45,46].

While Equation 4 produces a network that exhibits attractors, it is inconsistent with biology in at least two important ways. First, the neurons can exhibit both excitatory and inhibitory connections (for fixed presynaptic neuron *j*, *A_ij_* can be positive for some postsynaptic targets *i* and negative for others), which violates Dale's law. Second, connectivity is all to all, which is inconsistent with the sparse connectivity seen in cortex [47]. Both can be fixed by introducing sparse, random background connectivity among excitatory and inhibitory neurons, and adding a threshold so that neurons are either excitatory or inhibitory, but not both. This yields a set of connection strengths of the form

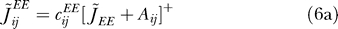


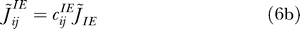


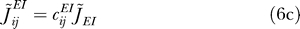


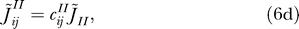
where the 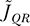 set the background connection strengths (with, of course, 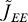 and 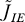 positive and 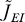 and 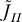 negative), [·]^+^ is the threshold-linear operator ([*x*]^+^= *x* if *x* > 0 and 0 otherwise), and 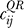 tells us whether neuron *j* of type *R* is connected to neuron *i* of type *Q*. We assume that the connection probability is independent of type, so

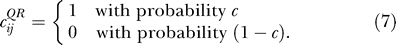
With this connectivity matrix, every neuron in the network projects to, on average, *K_E_* excitatory and *K_I_* inhibitory neurons, and every neuron receives, on average, *K_E_* excitatory and *K_I_* inhibitory connections, where

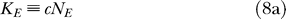


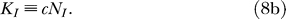



The probability of connection, *c*, is assumed to be much smaller than 1, leading to a sparsely connected network [47], and it is independent of the size of the network unless otherwise stated. While we could have made the connectivity scheme more general by letting the connection probability between neurons depend on their type and/or by letting the nonzero 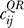 in Equation 7 have some variability, this would merely add complexity without changing any of our conclusions.

Although we are including the threshold-linear operator in Equation 6 (and also in the simulations), we neglect it in the forthcoming theoretical analysis. This is because *A_ij_* tends to be small: its mean is zero and, as we discuss in the sections “Storage capacity” and “Mean-field equations,” its variance is *O*(*p*/*K_E_*). Thus, as long as *p* is sufficiently small compared with *K*, the threshold-linear operator can be neglected. For our model, we find that *p*/*K* is at most about 0.01, which means that the threshold-linear operator is unlikely to have much effect. Importantly, even if *p*/*K* were large, the scaling relation that we derive for storage capacity, i.e., *p_max_* ∝ *K*, would still be correct; the only effect would be a slight modification to the precise value of *p_max_*/*K* [16].

### Network Equilibria

As discussed above, much of our focus in this paper is on solving Equation 3. For even moderate size networks, this corresponds to solving thousands of coupled, highly nonlinear equations, and for large networks that can number into the millions. We do not, therefore, try to find a particular solution to this equation, but instead look for a statistical description—a description in terms of probability distributions over excitatory and inhibitory firing rates. The main tool we use is self-consistent signal-to-noise analysis [48,49]. The idea behind this analysis is to treat the synaptic input (*h_Ei_* and *h_Ii_* in Equation 3) as Gaussian random variables. Solving Equation 3 then reduces to finding, self-consistently, their means and variances.

Because *h_Ei_* and *h_Ii_* consist of 2*K* (very weakly) correlated terms, where

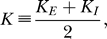
naive central limit arguments tell us that the standard deviations of these quantities should be smaller than their means by a factor of *K* ^1/2^. It would seem, then, that in the kinds of high connectivity networks found in the brain, where *K* is on the order of 5,000–10,000, neuron-to-neuron fluctuations in firing rate would be small, on the order of *K* ^−1/2^. By the same reasoning, *temporal* fluctuations in the firing rates would also be small, again on the order of *K* ^−1/2^. Neither of these, however, are observed in biological networks: there are large fluctuations in firing rate both across neurons and over time [24,50–53].

To resolve this apparent contradiction, one need only notice that *h_Ei_* and *h_Ii_* consist of both positive and negative terms (the first and third terms in Equation 2 are positive; the second is negative). If these terms approximately cancel—to within *O*(*K* ^−1/2^)—then both the mean and standard deviation of the synaptic drive will be on the same order, and network irregularity will be restored. As showed by van Vreeswijk and Sompolinsky in a groundbreaking set of papers [25,54], under fairly mild conditions this cancellation occurs *automatically*, thus placing networks very naturally in what they called the *balanced* regime. In this regime, fluctuations across both neurons and time are large. Whether networks in the brain really operate in the balanced regime is not completely clear, although recent experimental evidence has come down strongly in favour of this hypothesis [55,56].

While the work of van Vreeswijk and Sompolinsky was extremely important in shaping our understanding of realistic recurrent networks, their focus was primarily on random connectivity. The situation, however, is more complicated in attractor networks. That's because these networks consist of three classes of neurons rather than two: background excitatory neurons and background inhibitory neurons, as found in randomly connected networks, but also foreground excitatory neurons. Our goal in the next several sections is to understand how all three classes can be balanced, and thus fire irregularly.

#### Strong synapses and the balanced condition.

A reasonable constraint to place on our theoretical framework is that, in the large *K* limit, our results should be independent of *K*. This suggests that the synaptic strength, the 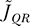 in Equation 6, should scale as *K* ^−1/2^. With this scaling, the mean value of the positive and negative terms in *h_Ei_* and *h_Ii_* become *O*(*K* ^1/2^); with cancellation these terms are *O*(1), and the variance is also *O*(1). Thus, if the gain functions, the *F_Q_*(*h*) in Equation 3, are also *O*(1), our results will be independent of the number of connections. To make the *K* ^−1/2^ scaling explicit, we define a new set of synaptic strengths and external input, which we denote *J_QR_* and *h_Qex_*, respectively,

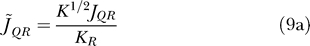


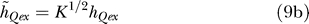
where *J_QR_* and *h_Qex_* are both *O*(1) and, recall, *K_R_* = *cN_R_* (Equation 8).

Equation 9 tells us how to scale the background connectivity, but it does not directly apply to the part of the connection matrix associated with memories, *A_ij_*. To determine how *A_ij_* should scale, we need only note that the mean contribution from the memories should be *O*(1)—sufficiently large to have an effect, but not so large as to overwhelm the background. Consequently, *A_ij_* should scale as 1/*K* (see the section “Mean-field equations” for details), which we can guarantee by defining a new variable, *β*, via the relation

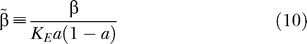
where *β* is *O*(1) and the factor *a*(1−*a*) is for convenience only.

#### Mean-field equations for the retrieval states.

Now that we have the “correct” scaling—scaling that makes our results independent of network size and ensures that the mean and variance of the synaptic input are both *O*(1)—we can apply self-consistent signal–noise analysis to Equation 3. The first step is to divide the excitatory and inhibitory synaptic currents (*h_Ei_* and *h_Ii_*) into two pieces: one that is nearly independent of index, *i* (the “mean”), and one that is a random variable with respect to *i* (the fluctuating piece). To do that, we rewrite the synaptic current in terms of our new variables, *J_QR_* and *β*, rather than 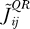 and 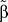 . Combining Equations 4, 6, 9, and 10 with Equation 2, we have

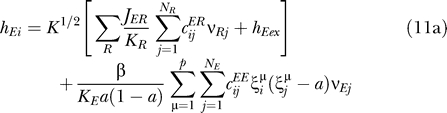


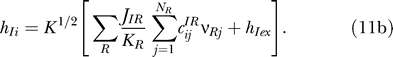
Note that Equation 11 is identical to Equation 2; it is just expressed in different variables.

For the terms in brackets, the mean and fluctuating pieces are easy to compute: the mean comes from replacing 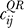 by its average, *c*, and the fluctuating piece comes from replacing 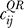 by the residual, 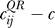 . For the second term in Equation 11a, separating the mean from the fluctuating piece is harder, as there is a nontrivial dependence on *i* associated with the *p* memories. Ultimately, however, we are interested in the case in which only one memory is retrieved, so when computing the mean we can consider only one term in this sum on *μ*; the other *p* − 1 terms contribute only to the fluctuations. Assuming, without loss of generality, that the first memory is retrieved, averaging over the randomness associated with the sparse connectivity allows us to replace 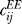 with *c*, and we find that the mean of the last term in Equation 11a is proportional to 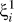 .

Putting all this together, we arrive at the eminently reasonable result that the mean excitatory and inhibitory synaptic currents are linear in the mean excitatory and inhibitory firing rates, and the mean excitatory current has an extra, memory-induced, dependence proportional to 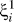 . Dropping the superscript “1” (a step taken only to simplify the equations), we find that the synaptic current may be written

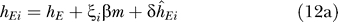


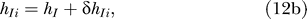
where *h_E_* and *h_I_* are the averages of the terms in brackets on the right-hand side of Equation 11a, *ξ_i_βm* is the mean contribution from the first memory, and 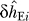 and *δh*_I*i*_ contain everything else. More specifically, the terms in Equation 12a are as follows. First, *h_E_* and *h_I_* are given by

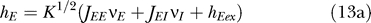


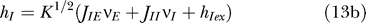
where *ν*_E_ and *ν*_I_ are the firing rates averaged over the excitatory and inhibitory populations, respectively,

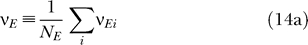


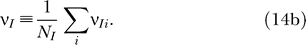



Second, the overlap, *m*, which is proportional to the mean firing rate of the foreground neurons relative to *ν_E_*, is given by

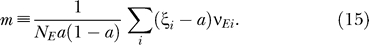



Expressions for the fluctuating terms, 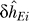 and *δh_Ii_*, are given in Equations 41 and 42. Because these terms contain *everything* not contained in the mean terms, Equation 12 is exact.

The three quantities *ν_E_*, *ν_I_*, and *m* are our main order parameters. To determine their values self-consistently, we express the firing rates, *ν_Ei_* and *ν_Ii_*, in terms of the synaptic currents using Equation 3, and insert those expressions back into Equations 14 and 15; that leads to

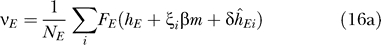


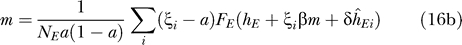


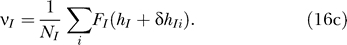



To solve these equations, we use the fact that there are a large number of neurons; this allows us to turn the sum over *i* into an integral over the probability distributions of 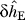 and *δh_I_*, denoted 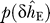 and 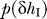 , respectively. Replacing the sum by an integral in Equation 16, and also averaging over ξ*_i_*, the mean-field equations become

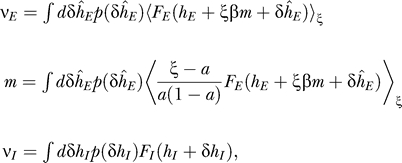
where the subscript on the angle brackets indicates an average with the statistics given in Equation 5.

Because both 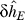 and *δh_I_* are Gaussian random variables (see the section “Fast fluctuations”), these integrals are reasonably straightforward; what makes them at all difficult is that the variance of 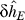 and *δh_I_* must be found self-consistently. This results in two more equations, for a total of five (see Equation 47). This is still far fewer than our original set of thousands or more. And the situation gets even better: it turns out that we really only need to consider three, at least if all we want to do is gain qualitative insight into how attractor networks function. That's because the integrals are simply Gaussian convolutions, so all they do is smooth the gain functions. Using a bar to denote the smoothed functions, and performing the average over *ξ* (which is straightforward because it has simple 0/1 statistics; see Equation 5), we have

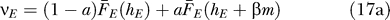


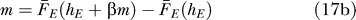


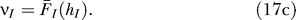



These equations—which are identical in form to the ones derived in [23]—are oversimplified versions of the full mean-field equations. Basically, the bar over *F* hides a dependence on two additional order parameters—the second moments of the excitatory and inhibitory firing rates—which in turn depend on our main order parameters, *ν_E_*, *ν_I_*, and *m*. While these dependencies are important for making detailed predictions, for an intuitive picture of what the mean-field equations mean they can be ignored. Consequently, in the next several sections, we focus on Equations 17a–17c, which we refer to as the reduced mean-field equations. At the end of the next section, we argue that, under very general conditions, all the conclusions we draw based on the reduced mean-field equations apply to the full set (which are given in Equation 47).

#### Reduced mean-field equations in the infinite *K* limit.

When solving the reduced mean-field equations, we have a choice: we can think of them as functions of *ν_E_*, *ν_I_*, and *m*, or as functions of *h_E_*, *h_I_*, and *m*. Since *ν_E_* and *ν_I_* are related to *h_E_* and *h_I_* via an invertible transformation—Equation 13—the two prescriptions are identical. The latter, however, turns out to be more convenient, especially in the infinite *K* limit. To see why, we need only solve Equation 13 for the mean firing rates, which yields

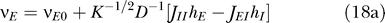


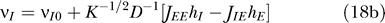
where

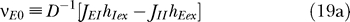


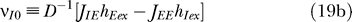
are the mean firing rates in the infinite *K* limit and

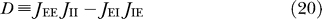
is the determinant of the background connectivity matrix; as shown in [54] and the section “Stability analysis,” *D* must be positive for the background to be stable. Since we are in the balanced regime, *h_E_* and *h_I_* are *O*(1). Consequently, in the infinite *K* limit, the mean excitatory and inhibitory firing rates are simply given by *ν_E0_* and *ν_I0_*, respectively, independent of *h_E_* and *h_I_*. Using this fact, the reduced mean-field equations, Equation 17, become, in the *K* → ∞ limit,

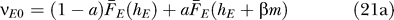


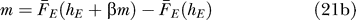


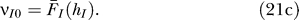



An important feature of these equations is that *h_I_* decouples from *h_E_* and *m*. This greatly simplifies the analysis, since it means we can find the equilibrium value of *h_I_* simply by inverting 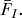


Our approach to finding the equilibrium values of *h_E_* and *m* is a graphical one: we plot, in *h_E_* − *m* space, the two curves that correspond to the solutions to Equations 21a and 21b—the *h_E_* and *m* nullclines, respectively—and look for their intersections. The goal is to determine the conditions under which there are multiple intersections, with at least one of them corresponding to an equilibrium with *m* > 0, and thus to a retrieval state.

**Figure 1:**
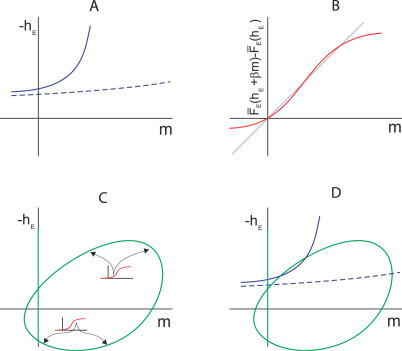
Generic Shapes of the Nullclines Note that these are “cartoons,” and thus do not apply to any particular model; for nullclines derived from a specific model, see Figure 2. (A) *h_E_*-nullcline versus *m* for two different values of *a* (*a* is small for the dashed curve and large for the solid curve). Note that we use −*h_E_* on the *y*-axis, so the upward curvature indicates that the total synaptic drive to a cell decreases with *m*. (B) Right-hand side of Equation 21b versus *m* with *h_E_* fixed. The intersections with the 45° line correspond to points on the *m*-nullcline. (C) The *m*-nullcline. The precise shape isn't so important; what is important is that the part of the nullcline not on the *m* = 0 axis has the topology of a circle. Insets indicate the portion of *F_E_*(*h_E_*) that contributes to the *m*-nullcline; see text. (D) The *m*- and *h_E_*-nullclines on the same plot. The intersections correspond to network equilibria. There are three equilibria: one at *m* = 0, corresponding to the background state, and two at *m* > 0, corresponding to potential retrieval states. The one at *m* = 0 and the one at large *m* are stable; the intermediate one is not. Consequently, only the large *m* equilibrium is observed during retrieval. Note that when the coding level, *a*, is small (dashed blue line), the retrieval state occurs at large *m*, and thus has a high firing rate. Only when *a* is large (solid blue line) is it possible to have a low firing rate during retrieval.

To be as general as possible, we make only two assumptions: 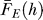 is monotonicaly increasing, and it is quasi-sigmoidal, where we use “quasi-sigmoidal” to mean convex (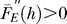 ) for small *h* and concave (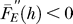 ) for large *h*. (Note that 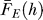 need not saturate.) This immediately tells us something about the shape of the *h_E_*-nullcline: since the right-hand side of Equation 21a is an increasing function of both *h_E_* and *m*, its solution, *h_E_*(*m*), must have negative slope (i.e., *dh_E_*/*dm* < 0 along the *h_E_* nullcline). Typical plots of the *h_E_*-nullcline are shown in Figure 1A for two values of the coding level, *a*. Note that the nullcline curves upward in this plot, a consequence of the fact that we use −*h_E_* rather than *h_E_* on the *y*-axis.

**Figure 2:**
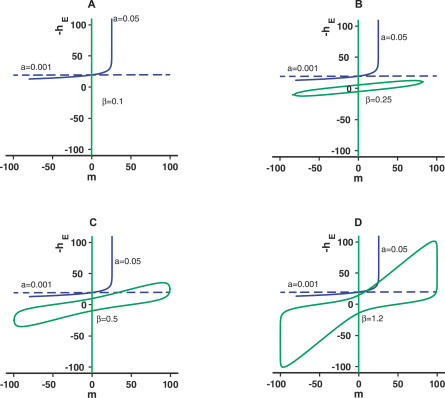
*h_E_*-Nullcline and *m*-Nullcline for the Gain Function given in Equations 23 and 24 Different panels correspond to different values of *β*, and in all of them two *h_E_*-nullclines are shown: one with *a* = 0.001 (dashed blue line) and one with *a* = 0.05 (solid blue line). The *m*-nullcline does not depend on *a* (Equation 21b). The parameters were *J_EE_* = *J_IE_* = 1, J*_EI_* = −1.9, *J*_II_ = −1.5, *h_Eex_* = 3, *h_Iex_* = 2.1, which implies, via Equations 19 and 20, that *ν*_*E0*_ = 1.3 Hz. (A) *β* = 0.1. The *m*-nullcline consists only of a line at *m* = 0, so there can be no memory retrieval states. (B) *β* = 0.25. The *m*-nullcline gains a second piece away from *m* = 0, but there are still no equilibria with nonzero *m*, and thus no retrieval states. (C) *β* = 0.5. The *m*-nullcline now intersects one of the *h_E_*-nullclines—the one with small coding level, *a*. (D) *β* = 1.2. There are now three intersections for both values of *a*. The ones with *m* = 0 and large *m* are stable; the one with intermediate *m* is unstable (see the section “Stability analysis”). The *h_E_* -nullcline with *a* = 0.001 is essentially a straight line, so memory retrieval occurs at a firing rate that is too high to be biologically realistic. The *h_E_*-nullcline with *a* = 0.05, on the other hand, has strong upward curvature, so memory retrieval occurs at a much lower, and thus biologically plausible, firing rate.

To find the *m*-nullcline—the set of points in *h_E_* − *m* space that satisfy Equation 21b—we proceed in two stages. First, we plot the right-hand side of Equation 21b versus *m* and look for intersections with the 45° line; these intersections correspond to points on the *m*-nullcline. Second, we vary *h_E_* and sweep out a curve in *h_E_* − *m* space; this curve is the full *m*-nullcline. A typical plot versus *m* with *h_E_* fixed is shown in Figure 1B. There are three intersections with the 45° line, which means that the *m*-nullcline consists of three points at this particular value of *h_E_*: one with *m* = 0 and two with *m* > 0. To find out how these three points move as we vary *h_E_*, we compute *dm*(*h_E_*)/*dh_E_* where the derivative is taken along the *m*-nullcline; using Equation 21b, this is given by

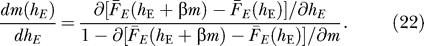



We are primarily interested in the sign of *dm*/*dh_E_*, which can be found by examining the signs of the numerator and denominator separately. For the denominator, note that the derivative of the term in square brackets is the slope of the curve in Figure 1B. Consequently, the denominator is negative for the intermediate intersection (where the slope is greater than 1) and positive for the upper intersection (where the slope is less than 1). The sign of the numerator depends primarily on the size of *h_E_*. If *h_E_* is small, so that both 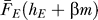 and 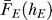 lie on the convex part of the sigmoid, then the numerator is positive. If, on the other hand, *h_E_* is large, so that 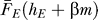 and 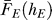 lie on the concave part, then it is negative (see insets in Figure 1C).

This gives us the following picture: when *h_E_* is small, so that the numerator in Equation 22 is positive, decreasing *h_E_* causes the two intersections in Figure 1B to move closer, and eventually to annihilate. When *h_E_* is large, on the other hand, so that the numerator is negative, increasing, rather than decreasing, *h_E_* causes the intersections to move closer, and eventually annihilate, this time for sufficiently large *h_E_*. Filling in the points away from the extrema, we see that the *m*-nullcline is topologically equivalent to a circle (Figure 1C). Finally we note that the line *m* = 0 is also part of the nullcline, as can easily be seen from Equation 21b; this line is also included in Figure 1C.

In Figure 1D, we combine the *h_E_*-nullclines from Figure 1A and the *m*-nullcline form Figure 1C. Clearly there is always an equilibrium at *m* = 0, corresponding to no active memories; i.e., corresponding to a null background. There are also two equilibria at *m* > 0, corresponding to active memories. In the section “Stability analysis,” we show that the one at larger *m* is stable. Importantly, this equilibrium can occur at small *m*, and thus low firing rate, something we will see more quantitatively in the next section, where we consider a specific example. Although not shown in Figure 1, the *m*-nullcline can shift far enough up so that *m* can be negative at equilibrium. When this happens, *m* = 0 becomes unstable, which in turn implies that the background becomes unstable. We see this in the simulations: when *β* becomes too large, memories are spontaneously activated.

We can now see the critical role played by the coding level, *a*. In the limit *a* → 0, the right-hand side of Equation 21a becomes almost independent of *m*. This makes the *h_E_*-nullcline almost horizontal (dashed line in Figure 1D), so the only stable retrieval state occurs at large *m*, and thus high firing rate (the peak of the *m*-nullcline typically occurs near the maximum firing rate of the neurons, about 100 Hz; see next section). If, on the other hand, *a* is reasonably large, then the *h_E_*-nullcline can curve up and intersect the *m*-nullcine to the left of its highest point (solid blue line in Figure 1D). As just discussed, this intersection corresponds to the intermediate intersection in Figure 1B, which means it corresponds to low firing rate, and thus a biologically realistic retrieval state.

We end this section by discussing the conditions under which the nullclines in Figure 1D, which were derived from Equation 17, are the same as the nullclines for the full mean-field equations, Equation 47. The primary effect of the full set of equations is to couple *h_I_* to *h_E_* and *m*. One could, however, solve for *h_I_* in terms of *h_E_* and *m*, insert that solution into the equations for *h_E_* and *m*, and derive a new coupled set of equations that again involve only *h_E_* and *m*. This would, effectively, replace 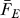 in Equation 17 with a more complicated function of *h_E_* and *m*. Examining Equations 47 and 48, we see that these manipulations would result in the following replacements,

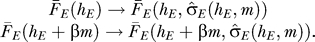
Retracing the steps that led us to Figure 1D, we see that if 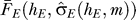 and 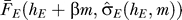 are quasi-sigmoidal functions of *h_E_* and *m*, we recover the nullclines in Figure 1D. Both of these conditions are likely to hold for real neurons, since increasing *h_E_* and *m* correspond to increasing excitatory drive. Thus, for neurons with reasonable gain functions, we expect Figure 1D to fully capture the shape of the nullclines.

#### An example: Nullclines for a simple gain function.

As an illustrative example, we consider a specific form for the gain functions (the 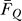 ), and compute the resulting nullclines numerically. The form we choose is a rather standard one,

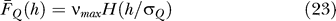
where *ν_max_* is the maximum firing rate of both excitatory and inhibitory neurons, which without loss of generality we take to be 100 Hz, H(*x*) is given by

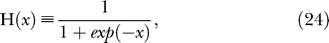
and *σ_Q_* is an approximate standard deviation based on Equation 44,

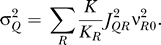



Before computing the nullclines for these gain functions, we introduce a transformation that changes the nullclines without changing the equilibria. Combining Equations 211a and 21b, we see that 21a can be written

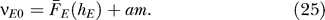



Note that the right-hand side of Equation 25 is an increasing function of both *h_E_* and *m*, so the *h_E_*-nullcline based on Equation 25 has the same qualitative shape as the *h_E_*-nullcline based on Equation 21a. This form is more useful than the one in Equation 21a, however, because we can immediately write down an expression for *h_E_*(*m*),

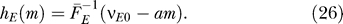



Computing the nullclines is now a straightforward numerical task, and in Figure 2A–2D we plot the *m*-nullclines (green) for increasing values of *β* and the *h_E_*-nullclines (blue) for two different values of the coding level, *a*. Because the *m*-nullcline does not depend on *a* (see Equation 21b), there is only one *m*-nullcline in each panel.

The first thing we notice is that when *β* is sufficiently small (Figure 2A), the *m*-nullcline consists only of a line at *m* = 0, which means that the only possible equilibria are at *m* = 0, and so retrieval states are not possible. When *β* is slightly larger (Figure 2B), the *m*-nullcline gains a second piece away from the line *m* = 0. However, this second piece lies below both *h_E_*-nullclines, so the only intersections are again at *m* = 0, and retrieval is again not possible. The fact that there is no memory retrieval when *β* is small makes sense: *β* controls the connection strength among the neurons within each memory, so if it is too small there will not be enough recurrent connectivity to produce elevated firing.

For still larger *β*, there is an intersection with one of the *h_E_*-nullclines—the one corresponding to low coding level (Figure 2C). The stable equilibrium, which is the equilibrium with larger *m*, corresponds to memory retrieval (see the section “Stability analysis”). Finally, at sufficiently large *β*, the system acquires an intersection with the *h_E_*-nullcline corresponding to high coding level (Figure 2D). Again, the stable equilibrium is the one with larger *m*.

An important point is that the value of *m* at the retrieval state, and thus the firing rate of the foreground neurons, depends strongly on the coding level, *a*. For small *a* (dashed blue line), retrieval occurs near saturation, and thus at an unrealistically high firing rate. For larger *a* (solid blue line), the retrieval occurs at low firing rate, consistent with experiments (when *a* = 0.05 and *β* = 1.2, the equilibrium value of *m* is 20 Hz). This is exactly the behaviour we saw in the previous section.

**Figure 3:**
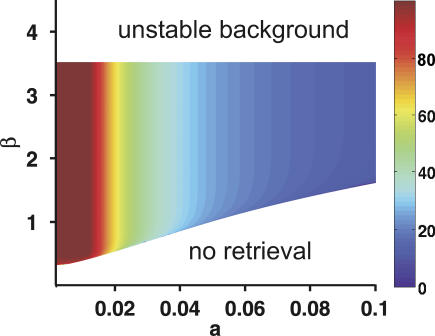
Phase Diagram Showing the Values of *a* and *β* Which Exhibit Both a Stable Background and Memory Retrieval The firing rate (in Hz) of the foreground neurons is indicated by the color bar on the right. Below the colored region, only the background exists, and it is stable. Above the colored region, the background is unstable. The upper boundary is defined through Equation 27; the lower boundary is determined numerically by finding the minimum value of *β* (for a given *a*) such that the *m*-nullcline and *h_E_*-nullcline intersect. The parameters are the same as in Figure 2: *J**_EE_*** = *J_IE_* = 1, *J_EI_* = −1.9, *J_II_* = −1.5, *h_Eex_* = 3, *h_Iex_* = 2.1.

As can be expected from these figures, increasing *β* even further would shift the intermediate intersection to negative values of *m*. In this regime the background becomes unstable. Again this makes sense: if the coupling among the neurons within a memory is too strong, they become spontaneously active. Examining Figure 1B, we see that this occurs when the slope of 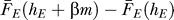 with respect to *m* is 1 at *m* = 0 (and, of course, *h_E_* is at its equilibrium value). The value of *β* at which this happens, denoted *β_max_*, is given by

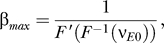
(see Equations 21b and 26). For the sigmoidal gain function used in this example (Equation 24), *β_max_* is given by

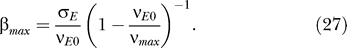
The phase diagram for this model—a plot showing stability and, in the stable region, the firing rate of the foreground neurons—is shown in Figure 3.

#### Storage capacity.

In the above analysis, there was no way to determine how many memories could be embedded in a network, and thus no way to determine storage capacity. That's because we hid all effects of the quenched noise—the noise associated with the random elements of the connectivity matrix—in 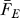 and 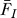 (see Equation 17). However, the quenched noise can have a nontrivial effect, in two ways. First, within the context of the self-consistent signal-to-noise analysis, it changes both 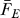 and 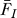 , and thus modifies the nullclines. Second, and potentially more important, as we add memories we increase the number of preferred modes that can be activated in the network, and thus we increase the quenched noise. Either effect could cause memories to be active when they should not be, and inactive when they should be.

To quantify these effects, we note that both scale with the fluctuations associated with the memories that are *not* recalled on any particular trial. The size of these fluctuations can be found by computing the contribution of the memories to 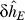 , the fluctuating piece in Equation 12a. Examining the memory portion of the connectivity matrix, *A_ij_*, which is given in Equation 4, and noting that 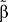 is proportional to 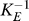 (Equation 10), we show in the section “Mean-field equations” that the variance of the quenched fluctuations associated with this term scale as *p*/*K_E_* (Equation 45). Intuitively, that is because when we sum the right-hand side of Equation 4 on *j* and *μ*, there are (*p* − 1)*K_E_* terms: *K_E_* that come from the sum on *j*, and *p* − 1 that come from the non-activated memories in the sum on *μ*. Each of these terms has variance that is 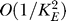 . Central limit type arguments then tell us that the variance of such a sum is on the order of 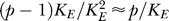 , where the approximation is valid if *p* is large. Consequently, there is a critical value of *p*/*K_E_* above which none of the stored patterns could be retrieved. Thus, the maximum number of memories in a network should scale linearly with *K_E_*. This is what we found in our simulations (see the section “Computer Simulations”).

Unfortunately, the scale factor we found in our simulations was small, in that the maximum number of memories scaled as 0.01 *K_E_*. A natural question to ask, then, is: can the scale factor be improved by, for example, using different parameters in our network? In the rest of this section, we focus on the effect of the coding level, *a*, on the storage capacity. We choose the coding level because, at least in simple models, the storage capacity is inversely proportional to *a* [33,34,37]. We have already shown that as the coding level decreases, the foreground firing rate becomes large, so we cannot make *a* arbitrarily small. However, the minimum allowable value of *a* depends on the model. What we show below, though, is that even for models which exhibit realistic foreground firing rate at relatively low coding levels, the 1/*a* scaling of the storage capacity does not hold. This suggests that decreasing the coding level cannot be used to increase the storage capacity in realistic networks.

**Figure 4:**
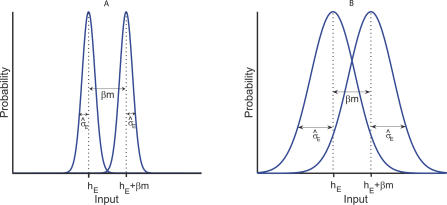
Distribution of Inputs to Foreground (Mean = *h_E_*) and Background (Mean = *h_E_* + *βm*) Neurons, and Its Relation to Storage Capacity Both inputs have a Gaussian distribution. The means are separated by *βm* and the standard deviation of both distributions is 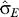 . (A) The standard deviation is much smaller than the distance between the means of the two distributions. In this regime, the two populations are well separated, there is no interference between them, and memory retrieval is supported. (B) As *α* increases, 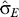 also increases (47d) while *m* changes rather slowly (47b), so the distributions start to overlap. When the overlap becomes large, noise dominates the signal, and memory recall is no longer possible. The value of *α* at which this happens is the storage capacity, *α_max_*.

Examining Equations 12a and 46, we see that the background neurons receive an input drawn from a Gaussian distribution with mean *h_E_* and standard deviation 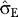 , while the foreground neurons receive input with larger mean, *h_E_* + *βm*, and the same standard deviation, 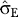 . When the standard deviation of these distributions, 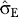 , is smaller than the separation between the means, the two populations are well separated (Figure 4A) and memory recall is possible. The standard deviation, however, is an increasing function of *p*; see 47d and note that *p* enters this equation only through the *storage load*, *α*, which is defined to be

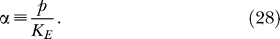
When *α*, and thus *p*, becomes large enough, the standard deviation is on the same order as the separation. At this point, the two distributions have a significant overlap with each other (Figure 4B), and memory recall fails.

Using this intuitive picture and Equation 47d, we can find the value of *α* for which 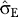 is on the order of the separation between the means; this should give us an estimate of the storage capacity, *α_max_*. Using Equation 47d and the fact that the means are separated by *βm* (see Figure 4), we see that this happens when

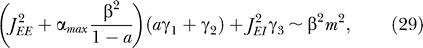
where

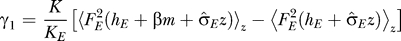


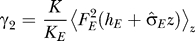


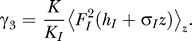



Solving Equation 29 for *α_max_* then leads to

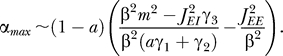
If the background synaptic weights, *J_EE_* and *J_EI_*, were zero and there was zero background firing so that *γ*_2_ vanished, we would recover the 1/*a* scaling (in the small *a* limit) found in simpler models [33,3437]. With nonzero background synaptic weights, however, the capacity no longer scales as 1/*a*. Consequently, we expect that the maximum capacity cannot be improved much by using sparser codes.

### Computer Simulations

Our mean-field analysis gave us two predictions. The first is that if the background synaptic weights, the 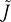 , scale as *K* ^−1/2^, the foreground weights, *A*, scale as *K* ^−1^, and the coding level, *a*, is sufficiently high, then both the background and foreground neurons should operate in the balanced regime and the neurons should fire irregularly. The second prediction is that the number of memories that can be stored is proportional to the number of excitatory connections per neuron, *K_E_*.

To test these predictions, we perform simulations with large networks of spiking neurons. We start by finding, for a particular network size, parameters such that both foreground and background neurons exhibit irregular activity. We then increase the size of the network while scaling the synaptic weights according to the above prescriptions. If the larger networks continue to exhibit irregular activity, then our predicted scalings are correct. To test the relation between storage capacity and number of connections per neuron, we calculate the storage capacity for networks with different sizes. A linear relation would indicate a scaling consistent with our predictions.

#### Network model.

Each neuron is modeled as a conductance-based quadratic integrate and fire (QIF) neuron. Dendritic trees and axonal arborizations are not considered. The spikes generated in any neuron immediately affect all the postsynaptic neurons connected to it. The membrane potential of neuron *i* of type *Q*, denoted *V_Qi_*, evolves according to

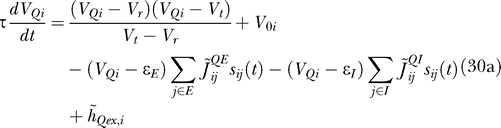


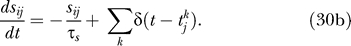
Here *τ* is the membrane time constant, *τ_s_* is the synaptic time constant, *V_r_* and *V_t_* are the nominal resting and threshold voltages, *V*_0*i*_ determines the actual resting and threshold voltages, (*V*_0*i*_ is constant for each *i*, but as a function of *i* it's a Gaussian random variable with mean *V*_0*i*_ and standard deviation Δ*V*_0_), 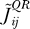 is the connection strength from cell *j* in population *R* to cell *i* in population *Q*, *ɛ_E_* and *ɛ_I_* are the excitatory and inhibitory reversal potentials, respectively, the notation *j* ∈ *R* means sum over only those cells of type *R*, *δ*(·) is the Dirac *δ*-function, 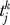 is the *k*th spike emitted by neuron *j*, and 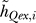 is the external input to neuron *i* of type *Q*. The external input is modeled as

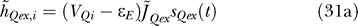


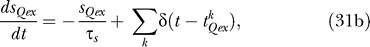
where the 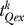 are the times of the external spikes. These are taken to be Poisson at constant rate *v_Qex_*.

There are two features of these equations that are worth commenting on. First, the connection strengths, 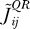 , are completely analogous to the ones given in Equation 2. Thus, although the 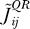 in Equation 30 have different numerical values than those in Equation 2, they should have the same scaling with connectivity [40,44,57]. The same is also true of 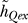 , except that here 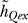 has temporal fluctuations whereas in Equation 2 it does not. Second, we have included a term *V*_0*i*_, which has the effect of making the resting membrane potential and threshold of each cell different. This was not explicitly modeled in our mean-field analysis, although it would not have made much difference—it would have only added to the quenched noise.

The 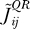 have the same form as in Equation 6, except that we introduce an extra scaling factor so that connection strengths can be directly related to postsynaptic potential (PSP) size. Specifically, we use the fact that if neuron *j* spikes and neuron *i* is at rest, then the PSP generated at neuron *i* will have peak amplitude 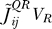 where

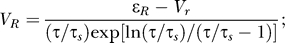
see [58] for a derivation of this expression. This suggests that we should scale our connection strengths by *V_R_*, so we write

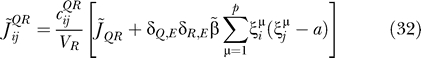
where 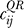 is the same binary random variable defined in Equation 7, *δ_Q_*_,*R*_ is the Kronecker delta, and 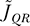 and 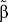 in Equation 32 correspond to, but typically have different numerical values than, the ones in Equations 6 and 10. If *V_R_* is in mV, then 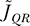 is the peak PSP, in mV, that occurs in a neuron in pool *Q* when a neuron in pool *R* fires (assuming the two are connected, the postsynaptic neuron is at rest, and 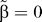 ).

Our analytical results have been derived by assuming current-based neurons. However, it is possible to extend such analysis to a more realistic network of conductance-based neurons by noting that the effective connection strength in a conductance-based model is proportional to the PSP size [40,44,57]. Thus, for the network to operate in the balanced regime, we should have the following scalings,

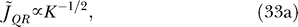


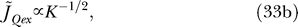


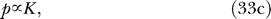


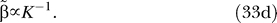
Note that the mean external excitatory input must be proportional to *K* ^1/2^. Therefore, given Equation 31a and the scaling of 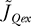 in Equation 33b, the firing rate of the neurons that provide external input, *v_Qex_*, should scale as *K*.

We performed simulations using three different networks, called Networks 1, 2, and 3, that differ in the number of neurons (they contain a total of 10,000, 20,000, and 30,000, respectively). In all three networks, *c* = 0.15, so *K* is proportional to the total number of neurons in the network. Because of the scaling in Equation 33, the values of 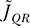 , 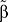 , *v_Qex_*, and *p* also differ. The parameters for the three networks are given in Table 1. Our goal in these simulations is to determine whether, as predicted by our mean-field analysis, the above scaling leads to behaviour that is independent of *K* and the firing of both foreground and background neurons is irregular.

**Table 1:**
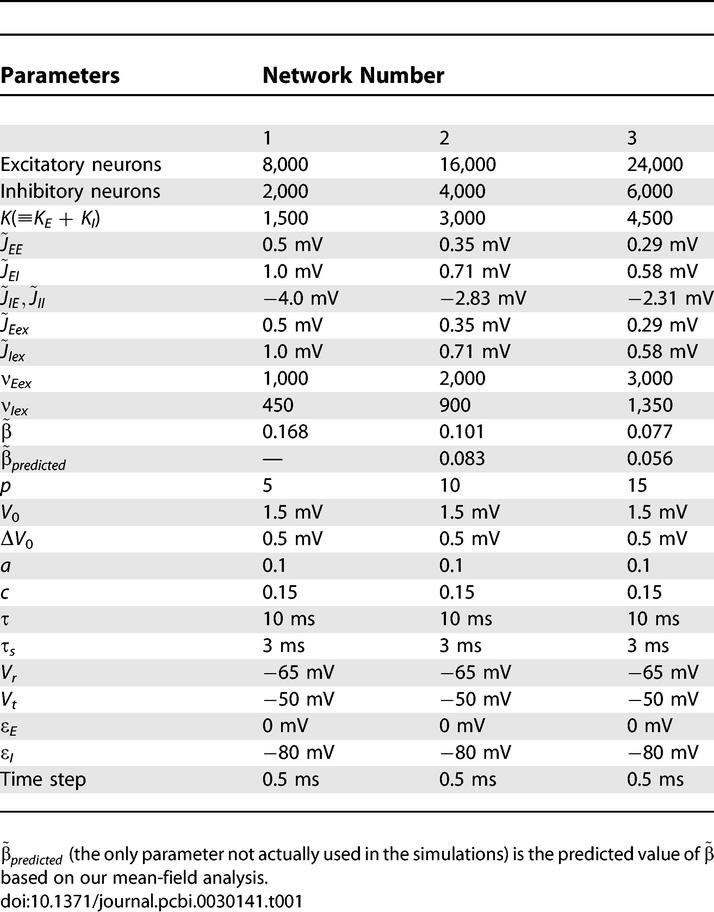
Parameters Used in the Simulations

#### Building a balanced network.

Our first step in assessing our mean-field predictions is to build a network that operates in the balanced regime and supports retrieval states. To test whether a network is operating in the balanced regime, we rely on two indicators. One is that it exhibits irregular firing, quantified by the coefficient of variation (CV)—the ratio of the standard deviation to the mean interspike interval—and that the CV is independent of *K*. The second is that the mean excitatory and inhibitory firing rates scale linearly with the external input, as predicted by Equation 19. To test whether a network supports retrieval states, we simply activate a memory by bombarding all the neurons within a memory with excitatory input, and ask whether the memory stays active for several seconds. Very little fine-tuning was required to find a network that exhibited both balance and retrieval states: we simply chose reasonable peak PSPs, set the coding level, *a*, to 0.1, and increased 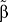 until at least one memory was stored.

**Figure 5:**
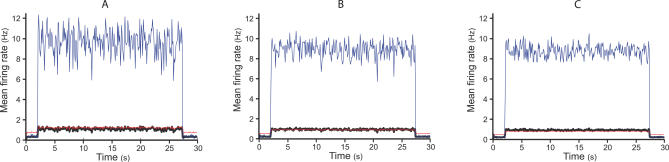
Examples of Activation of a Retrieval State (A) Network 1. (B) Network 2. (C) Network 3. Colors indicate mean population activity. Blue: foreground neurons. Black: excitatory neurons. Red: inhibitory neurons. At *t* = 2 s, neurons selective for one of the patterns receive a 100-ms barrage of excitatory input; at *t* = 27.3 s, the same neurons receive a barrage of inhibitory input.

In Figure 5A, we show an example of the retrieval of a stored pattern for Network 1. The first 2 s in this figure consists of background firing; at *t* = 2 s, neurons selective for one of the patterns receive an excitatory external input lasting for 100 ms; and at *t* = 27.3 s, the same neurons receive an inhibitory external input, which again lasts for 100 ms. The blue line is the mean firing rate of the foreground neurons, the black line is the mean firing rate of the excitatory neurons (both foreground and background), and the red line is the mean firing rates of the inhibitory neurons.

**Figure 6:**
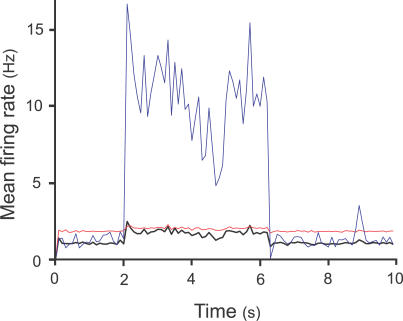
Retrieval States with Higher External Input Than in Figure 5, and thus Higher Background Firing Rate All parameters except *ν_Eex_*, *ν_Iex_*, *β*, and *p* are the same as in Network 1: here *ν_Eex_* = 5,000 Hz, *ν_Iex_* = 2,250 Hz, *β* = 0.325, and *p* = 1, versus Network 1, where *ν_Eex_* = 1,000 HZ, *ν_Iex_* = 450 Hz, *β* = 0.167, and *p* = 5. The stored pattern receives input for 100 ms, starting at *t* = 2 s, and then receives an external inhibitory current, again for 100 ms, starting at *t* = 6.2 s.

Two points are worth mentioning. One is that the background firing rate in our simulations is lower than the background firing rate observed in studies of delay activity, which range from 1.5 to 8 Hz [15], although we should point out that the firing rates determined from extracellular recordings may be overestimated due to selection bias [59]. We could, however, achieve a higher background rate by increasing the excitatory external input; an example is shown in Figure 6, for which the network parameters are the same as Network 1 (Figure 5A) except that the external input to excitatory and inhibitory neurons is five times higher, *β* is a factor of about two higher, and there is just one stored pattern instead of five. With the higher input, the background and foreground rates are in the range reported from neurons in, for example, anterior ventral temporal cortex [1,3] and entorhinal cortex [15].

The second point is that during retrieval, the mean firing rates of the excitatory and inhibitory neurons differ from the background rates; i.e., from the rates when no memories are activated. This appears to be inconsistent with the balance condition, which predicts that the mean firing rate during the activation of a memory is the same as that when the network is in the background state (see Equation 19). However, this prediction holds only in the limit of infinite connectivity. For finite connectivity, there are corrections, and they are particularly important when the firing rate is low [54]. For example, in Figure 5A the average excitatory activity increased from 0.28 Hz in the background to 1.07 Hz during retrieval (an increase of about 400%), whereas in Figure 6, where the background is higher, it increased from 1.06 Hz to 1.73 Hz (an increase of 60%). Thus, the increase in the mean excitatory firing rate during retrieval is reduced when the firing rate is higher. However, this is accompanied, at least in the parameter range we looked at, by a decrease in the storage capacity. Since we would like to study the scaling of storage capacity, we operate in the lower firing rate regime. A detailed search of parameter space is required to determine whether both high storage capacity and high background firing can be achieved.

**Figure 7:**
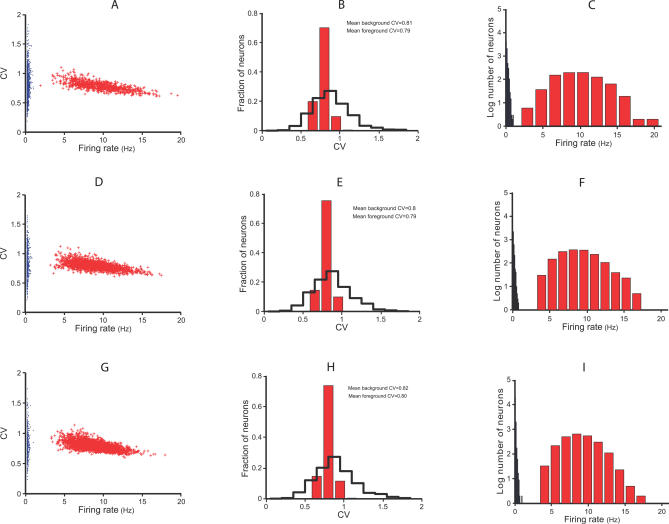
The Distribution of CVs and Firing Rates for Foreground and Background Neurons The first, second, and third rows correspond to Networks 1, 2, and 3, respectively. (Column 1) CV versus the firing rate of background (blue dots) and foreground (red crosses) neurons. Consistent with activation of a memory state, the neurons fall into two clusters, one corresponding to the foreground and the other to the background. (Column 2) Distribution of CVs for foreground (filled red bars) and background (solid line). The mean of both distributions is about 0.8, reflecting the fact that the neurons are firing irregularly. (Column 3) Distribution of firing rates for foreground (filled red bars) and background (solid line).

**Figure 8:**
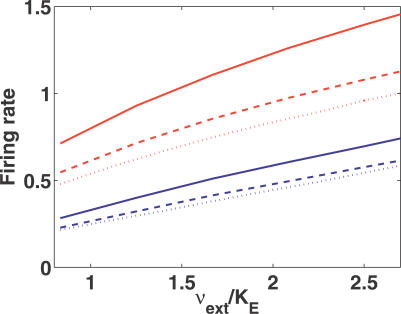
Average Excitatory (Blue) and Inhibitory (Red) Firing Rate versus External Input to Excitatory Neurons, Measured as Firing Rate per Connection (*ν_Eex_*/*K_E_*) The ratio *ν_Iex_*/*ν_Eex_* was fixed at 0.45. Full lines, dashed lines, and dotted lines correspond to Networks 1, 2, and 3, respectively. The average rates are calculated during a four-second period which consists of background firing only. The linear relationship between the mean inhibitory and excitatory firing rates and the external input is a signature of the balanced regime.

In Figure 7A we show the CV versus firing rate, again for Network 1. Here and in what follows, the CV is calculated only for those neurons that emit at least five spikes during the 25 s period that the pattern is active. The data in Figure 7A fall into two clusters, one (blue dots) corresponds to background neurons and the other (red crosses) to foreground neurons. The distributions of CVs and firing rates are shown in Figure 7B and 7C. The CV of both background and foreground neurons are on the order of 0.8, which indicates irregular firing. This suggests that the network is operating in the balanced regime. To further test for balance, in Figure 8A we plot the average excitatory and inhibitory firing rates versus the external input. As predicted by Equations 18 and 19, the relation is approximately linear.

#### Scaling of the parameters.

To test our predicted scaling with the number of connections, we considered networks with two and three times the number of neurons and connections as in Network 1; these are Networks 2 and 3. At the same time, we scaled 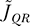 by 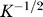 , ν*_Qex_* by *K*, and *p* by *K* (see Equations 33a–Equations 33c). The value of 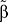 was set, as in Network 1, to the minimum value that results in retrieval of a single stored pattern. The 1/*K* scaling of *β* (Equation 10) gives us the values reported as 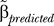 in Table 1. The values found from simulations (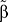 in Table 1) do not exactly follow the expected 1/*K* scaling: 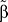 is 20% too large in Network 2 and 40% too large in Network 3. As discussed in the section “Retrieval states in the finite connectivity regime,” this is because of finite *K* effects, and the trends we see here follow the trends predicted in that section.

Examples of stored memories in Networks 2 and 3 are shown in Figures 5B and 5C, the CV versus firing rate is shown in Figures 7D and 7G, and the distribution of background and foreground CV and firing rates during the 25 s period that the memory is active are shown in Figure 7E and 7F for Network 2 and Figure 7H and 7I for Network 3. These plots show that when the connection strengths are scaled properly, both the background and foreground neurons exhibit irregular firing, just as in Network 1. Finally, Figure 8B and 8C show the relationship between the external input and the firing rate of the inhibitory and excitatory populations. As we saw for Network 1, the firing rate of excitatory and inhibitory neurons are linearly related to external input, further evidence for the balanced regime. In theory, the lines should lie on top of each other; however, due to finite size effects, this does not happen. The fact that finite size effects are responsible for this deviation from the theory can be seen by noting that the lines corresponding to Network 2 and Network 3 are much closer to each other than Network 1 and Network 2.

#### Scaling of the maximum number of memories.

Our last prediction is that the maximum number of memories should be linear in the number of excitatory connections, *K_E_*. To test this, for each of our three networks we increased the number of patterns, *p*, until the network failed to exhibit retrieval states. Specifically, we performed simulations as described in Figure 5, except that the memory was active for 4 s rather than 25 s. For each value of *p*, we activated, one at a time, either all *p* memories (if *p* was smaller than 20) or 20 memories (if *p* was larger). If the mean activity of the foreground neurons during the activation period was at least three times larger than the activity averaged over all the excitatory neurons, then that memory was said to be successfully retrieved.

**Figure 9:**
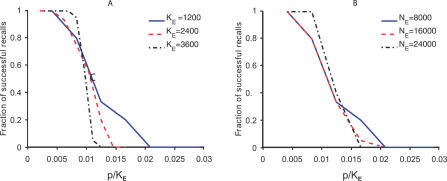
Scaling of the Maximum Number of Patterns with the Number of Excitatory Connections per Neuron, *K_E_* (A) The fraction of successful runs versus the storage load, *α* = *p*/*K_E_*, for three different values of *K*_E_. The size of the network is scaled such that we always have *K_E_*/*N_E_* = *K_I_*/*N_I_* = 0.15. There is a critical value of *α*, above which the fraction of successful runs is zero; this is the storage capacity *α_max_*. The transition at *α_max_* is sharp for *K_E_* = 3,600 but smoother for *K_E_* = 2,400 and *K_E_* = 1,200, due to finite size effects. The fact that *α_max_* is almost the same for all three values of *K_E_* implies that the maximum number of patterns that could be stored and retrieved, *p_max_*, is linear in *K_E_*. (B) The fraction of successful runs versus the storage load, *α* = *p*/*K_E_*, for three networks with all parameters, except for the total number of neurons in the network, is equal to those of Network 1. This figure shows that increasing the size of the network does not change *p_max_*.

The results of these simulations are shown in Figure 9A, where we plot the fraction of successful retrievals versus *p*/*_KE_* for the three networks. Consistent with our predictions, the transition to a regime where none of the patterns could be retrieved occurs at approximately the same value of *p*/*_KE_* for all three networks. Moreover, as one would expect, the transition for the largest network is sharper than for the others.

Although Figure 9A shows that *p_max_* scales linearly with *K_E_*, in these simulations *N_E_* also scales with *K_E_*, so this does not rule out the possibility that *p_max_* is proportional to *N_E_* rather than *K_E_*. To test for this, in Figure 9B we plot the fraction of successful retrievals versus *p*/*_KE_*, but this time with *K_E_* fixed and *N_E_* varied. This figure shows that *p_max_* is proportional to *K_E_*, not *N_E_*, ruling out the *N_E_* scaling.

## Discussion

In this paper we addressed two questions. First, can all the neurons in an attractor network—both background and foreground—exhibit irregular firing? And second, what is the storage capacity in networks of realistic spiking neurons? To answer these questions, we applied self-consistent signal-to-noise analysis to large networks of excitatory and inhibitory neurons, and we performed simulations with spiking neurons to test the predictions of that analysis.

Our primary finding is that two conditions must be met to guarantee irregular firing of both foreground and background neurons. The first is proper scaling with the number of connections per neuron, *K*: the strength of the background weight matrix must scale as *K* ^−1/2^ and the strength of the structured part of the weight matrix (the part responsible for the memories) as *K* ^−1^. What this scaling does is guarantee “balance,” meaning the network dynamically adjusts its firing rates so that the mean input to a neuron is on the same order as the fluctuations, independent of *K*. This in turn guarantees that the degree of irregular firing is independent of *K*.

While balance is a necessary condition for irregular firing, it is not sufficient. That's because balance ensures only that the mean and fluctuations are independent of *K*, but does not rule out the possibility that the mean is much larger than the fluctuations, which would result in regular firing. To ensure that this does not happen, a second condition must be satisfied: the coding level, *a*, must be above some (*K*-independent) threshold. This condition is needed to ensure that the coupling between background and foreground neurons is sufficiently strong to stabilize a low firing rate foreground state on the unstable branch of the *m*-nullcline (see Figure 1).

The analysis that led to predictions of irregular firing also quite naturally provided us with information about the capacity of attractor networks—the maximum number of patterns that could be stored and successfully retrieved. What we found, under very general conditions, was that this maximum, denoted *p_max_*, is linear in the number of excitatory connections per neuron, *K_E_*. This scaling relation has been observed in studies of simplified attractor networks [16,32,34], but, as discussed in the Introduction, those models did not include all the features that are necessary for a realistic recurrent networks. Thus, the analysis performed here is the first to show that the number of memories is linear in *K_E_* in biophysically plausible networks.

### Scaling in Other Models, and the Importance of *O*(1) Input to the Foreground Neurons

Note that there are other types of scaling, different from what we proposed, which can result in irregular firing of both foreground and background neurons. What is critical is that the net input a foreground neuron receives from the other foreground neurons should be *O*(1). We achieved this by letting the structured part of the connection matrix (the second term in Equation 11a) be *O*(1/*K*), and using a coding level, *a*, that was *O*(1). However, this is not the only possible combination of connection strengths and coding levels, and in the two other studies that address both scaling and irregularity in memory networks [27,28], different combinations were used. In the model proposed by van Vreesjwik and Sompolinsky [27], the structured part of their connection matrix was a factor of *K* ^1/2^ larger than ours; to balance that, the coding level was a factor of *K* ^1/2^ smaller. In the model proposed by Renart et al. [28], the structured part of the synaptic weights was *K* times larger than ours, so their coding level had to scale as *O*(1/*K*). Whether such low coding levels are consistent with reality needs further investigation; however, data from studies conducted on selectivity of neurons to visual stimuli suggests that it is too low [30]. In addition to the very low coding level that these two models require, they also exhibit non-biologically high foreground firing rate. Nevertheless, the model of Renart et al. [28] does have one advantage over others: the foreground neurons are as irregular as, or even more irregular than, the background neurons, something our model does not achieve (see next section).

### Not as Irregular as It Could Be

Although our simulations showed irregular activity, we found that the mean CV was only about 0.8. This is smaller than the values measured in vivo, which are normally close to, or slightly above, one [24,50–53]. In addition, in our simulations the CV showed a small, but consistent, decrease with firing rate (see the left column in Figure 7). This is due to the fact that with the scaling that we chose, the fluctuations in the input current to foreground and background neurons are the same but the mean current to the foreground neurons is higher (see the section “Fast fluctuations”). This decrease in the CV disagrees slightly with a study by Compte et al. [24], who found that the CV in prefrontal cortex does not depend on the mean firing rate, at least in a spatial memory task. While there are many possible reasons for this discrepancy, a likely one arises from the fact that the neurons in our network contained only two time scales, the membrane and synaptic time constants, and both were short: 10 ms for the former and 3 ms for the latter. Real neurons, however, have a host of long time scales that could contribute to irregularity [60]. In addition, in vivo optical imaging [61–63] and multi-electrode [64] studies indicate that the background activity varies coherently and over long time scales, on the order of seconds, something we did not model. Both of these would increase the CV, although how much remains to be seen.

Although multiple time scales could certainly increase irregularity, it is not the only possible way to do this. As discussed in the Introduction and in the previous section, the model proposed by Renart et al. [28] also increases irregularity, and is consistent with the experimental results of Compte et al. [24]. However, it requires a very small coding level (*a* ∝ 1/*K*), and fine-tuning of the parameters.

### Subthreshold versus Suprathreshold Persistent Activity

In conventional models of persistent activity [14,22,29], the foreground activity necessarily lies on the concave part of the excitatory gain function, *F_E_*(*h_E_*), whereas the background activity lies on the convex part. Since the inflection point of realistic gain functions is typically near the firing threshold [42,43], this type of bistability is called suprathreshold bistability [22,28]. Because the concave part of the gain function is typically at high firing rate, with suprathreshold bistability it is hard to have either low foreground firing rate or high CV. Consequently, there has been interest in understanding whether it is possible to have subthreshold bistability; that is, whether it is possible for both foreground and background solutions to lie on the subthreshold part of the gain function [28].

The model presented here can in fact show subthreshold bistability: as discussed in the section “Reduced mean-field equations in the infinite *K* limit,” increasing the coding level, *a*, brings the foreground firing rate very close to the background rate. Therefore, for sufficiently large *a*, the foreground state would be on the convex part of the transfer function. Our model, and the recently proposed model by Renart et al. [28], are the only ones that can show subthreshold bistability.

### Bimodal Distribution of Firing Rates

One rather striking feature of our networks is that they all produce a highly bimodal distribution of firing rates: as can be seen in the first and third columns of Figure 7, the background neurons fire at a much lower rate than the foreground neurons—so much lower, in fact, that they form a distinct, and easily recognizable, population. This occurs because the patterns we store—the 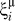 —are *binary*, which makes the average input current to every neuron in the foreground exactly the same. This feature is potentially problematic, as the distinction between foreground and background rates observed in experiments is not nearly as striking as the one in Figure 7 [65]. However, this feature is not essential to our analysis, for two reasons. First, as discussed in the section “Building a balanced network” (see especially Figure 6), we deliberately made the background firing rate low to increase the capacity. Second, it is easy to extend our analysis to real valued patterns in which the elements of the 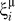 are drawn from a continuous distribution [34]. Under this, more realistic, scenario, it should be possible to match the statistics of the response seen in the cortex. This will be the subject of future work.

### Fine-Tuning of the Weights

In our model, every time a new pattern is learned, the weights change by an amount proportional to *K* ^−1^. This is a factor of *K* ^−1/2^ smaller than the background weights. Since weight changes are unlikely to be under such fine control, it is natural to ask whether errors during learning will lead to a major reduction in storage capacity. The answer, of course, depends on the size of the errors. In the section “Fine-tuning in the learning rule,” we show that errors can be larger than the weight changes by a factor of (*K*/*p*)^1/2^, with only a small change in storage capacity. More specifically, every time a pattern is learned, noise of *O*((*Kp*)^−1/2^) can be added to the synaptic strength, and the network will retain its ability to store and recall patterns.

Although this result tells us that the noise in the weight changes can be large compared with the structured part, the fine-tuning problem is not entirely eliminated: the noise must still be a factor of *p* ^1/2^ smaller than the background weights. Because of the low storage capacity found in these networks (at most 2.5% [23]), even when *K* is as large as 10,000, 1/*p* ^1/2^ is on the order of 6%. It seems plausible that biological machinery has evolved to achieve this kind of precision. However, for networks with larger capacity, the requirements on the precision of the weight would be more stringent.

It is also possible to have a probabilistic learning rule for which the changes in the weights are on the same order as the background weight, but this decreases the capacity significantly, by a factor of 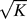 (see the section “Fine-tuning in the learning rule,” Equation 78; we thank Carl van Vreeswijk for pointing this out). Although this probabilistic learning rule guarantees a balanced state with irregular background and foreground firing, it has the drawback that the storage capacity scales as 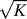 rather than *K*.

### Low Storage Capacity

Although we showed that *p_max_* ∝ *K_E_*, we did not compute analytically the constant of proportionality. In our simulations, this constant was small: from Figure 9, *p_max_* is about 0.01 *K_E_*, which means that for *K_E_* = 10,000 we can store only about 100 patterns. It is important, though, to note that we made no attempt to optimize our network with respect to other parameters, so the constant of proportionality 0.01 is unlikely to be a fundamental limit. In fact, Latham and Nirenberg [23] were able to store about 50 patterns in a network with 2,000 excitatory connections, 2.5 times larger than our capacity. Interestingly, the only substantial difference between their network and ours was that in theirs the background activity was generated by endogenously active neurons rather than by external input.

Can we further increase the scaling factor? One potential mechanism is to decrease the coding level, *a*, since, at least in simple models [33,34,37], the maximum number of patterns that could be stored and retrieved is inversely proportional to the coding level. But, as we showed in the section “Storage capacity,” realistic networks do *not* exhibit this 1/*a* scaling. Consequently, sparse coding cannot be used as a way to improve the storage capacity in our network. Simplified models also suggest that one can increase the storage capacity by a factor of 3–4 by using other schemes, such as non-binary patterns [34], or spatially correlated patterns [66]. Whether these techniques can be extended to the kind of network we have studied here is not clear, and requires further investigation. However, an increase beyond a factor of 3–4, to a capacity above about 0.1, seems unlikely within this class of networks.

In any case, there is a limit to the number of memories that can be stored in a single attractor network with a fixed number of connections per neuron, no matter how many neurons in the network. This suggests that, in order to make the best use of the existing connections, realistic working memory systems must be composed of interconnected modules. In this paradigm, each module would consist of an attractor network [67–69]. Such modular structure naively suggests a combinatorial increase in storage capacity; however, understanding how to achieve such an increase has proved difficult. For simple models whose storage capacity could be calculated analytically, either no increase in the storage capacity [67] or a modest increase [69] was found. It is yet to be determined how modular networks could be implemented in realistic networks of spiking neurons, and what their storage capacity would be.

## Materials and Methods

### Fast fluctuations.

The starting point for essentially all of our analysis is Equation 1, which, when combined with Equation 2, tells us that the time evolution of the firing rate of each neuron is purely a function of the firing rates of the other neurons. At a microscopic level, though, each neuron sees as input a set of spikes, not rates. However, for our model, rate-based equations do apply, as we show now.

In a spiking, current-based network, the input, *h_Qi_*(*t*), to the *i*th neuron in population *Q* has the form

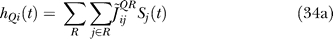


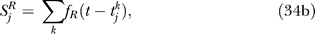
where 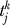 is the time of the *k*th spike on the *j*th neuron, *f_R_*(*t*), which mimics the PSP, is a non-negative function that integrates to 1 and vanishes for *t* < 0 and *t* large (greater than a few tens of ms). In a slight departure from our usual convention, *R* can refer to external input (*R* = *ex*) as well as excitatory and inhibitory input (*R* = *E*,*I*).

Our first step is to divide the input, *h_Qi_*, into a mean and a temporally fluctuating piece. The mean, which is found by time-averaging the right-hand side of Equation 34a and using the fact that *f*_R_(*t*) integrates to 1, is simply

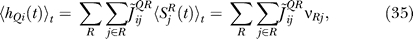
where 〈···〉*_t_* represents a temporal average. The temporally fluctuating piece of the input can then be written

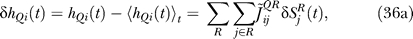


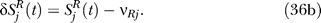
The fluctuations, *δh_Qi_*, have zero mean by construction, and their correlation function, *C_Qi_*(*τ*), is defined to be

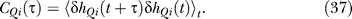



Assuming that *h_Qi_* is Gaussian (which is reasonable if there are a large number of neurons and they are not too correlated), then the firing rate depends only on the mean, 〈*h_Qi_*(*t*)〉*_t_*, and the correlation function, *C_Qi_*(*τ*). If the correlation function is independent of *i*, then the only *i*-dependence in the firing rate is through the mean input, and we recover Equation 1. What we now show is that, for our model, *C_Qi_* does not depend on *i*.

To understand the behaviour of *C_Qi_*, we express it in terms of 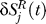 ; using Equation 36a, we have

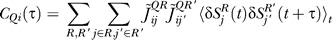
Under the assumption that the neurons are very weakly correlated, only the terms with *j* = *j′* survive, and this expression simplifies to

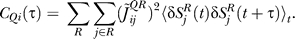



Let us focus on the sum on *j* on the right-hand side of this expression. For *Q ≠ E* or *R ≠ E*, this sum is given by (see Equations 6b–6d)

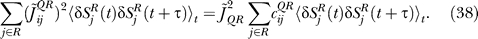
For sparsely connected networks, 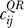 is independent of 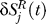 . Consequently, we can replace 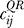 on the right-hand side of Equation 38 by its average, *c*, and the right hand side becomes independent of *i*.

For *Q* = *R* = *E*, the situation is more complicated, as 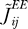 has an additional dependence on *A_ij_*, the structured part of the connectivity. Specifically using Equation 6a and again replacing 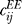 by its average, *c*, we have

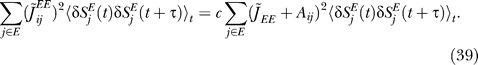
As discussed in the section “Mean-field equations,” *A_ij_* receives contributions from two sources: the *p* − 1 patterns that are not activated, and the one pattern that is. The non-activated patterns are not correlated with *δS_j_*, so they can be averaged separately in Equation 39, and thus do not produce any *i*-dependence. The activated pattern, on the other hand is correlated with *δS_j_*. However, the connection strength for the one activated pattern is smaller than 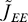 by a factor of *K* ^−1/2^ (see the section “Strong synapses and the balanced condition”). Consequently, in the high connectivity limit, we can ignore this contribution, and the right-hand side of Equation 39 is independent of *i*. This in turn implies that *C_Qi_* depends only on *Q*.

The upshot of this analysis is that the only *i*-dependence in the firing rate comes from 〈*h_Qi_*(*t*)〉*_t_*. Moreover, comparing Equations 2 and 35, we see that 〈*h_Qi_*(*t*)〉*_t_* is exactly equal to *h_Qi_*, the input current to the firing rate function, *F_Q_*, that appears in Equation 1. Thus, for the model used here, the rate-based formulation is indeed correct. What we do not do is compute *F_Q_*, as that would require that we compute the correlation function, *C_Q_*(*τ*), self-consistently, which is nontrivial [44]. However, our results depend very weakly on the precise form of *F_Q_*, so it is not necessary to have an explicit expression for it.

### Mean-field equations.

In this section, we derive the mean-field equations for the model described in the section “Model.” As discussed in the main text, the derivation of these equations revolves around finding the distributions of 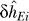 and *δh_Ii_*, the fluctuations around the mean excitatory and inhibitory synaptic input (both quantities are defined implicitly in Equations 11–13). The main assumption we make is that 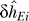 and *δh_Ii_* are zero mean Gaussian random variables, so all we need to do is find their variances self-consistently. In addition, primarily for simplicity (and because it is reasonable in large networks in the brain), we assume that the number of connections is small compared with the number of neurons, so *c* ≪ 1.

Our first step is to simplify the expressions for our main order parameters, *ν_E_*, *m*, and *ν_I_*. In the context of the self-consistent signal-to-noise analysis, “simplify” means “replace sums by Gaussian integrals.” To see how to do this, note that, for any function *g*,

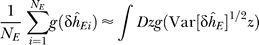
where Var[·] indicates variance, exact equality holds in the *N_E_* → ∞ limit (but approximate equality typically holds when *N_E_* is only a few hundred), and

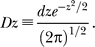
A similar expression applies, of course, to *δh_Ii_*.

Applying the sum-goes-to-integral rule to Equation 16, we have

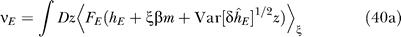


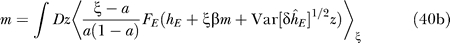


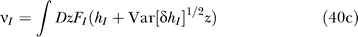
where the average over *ξ* is with respect to the probability distribution given in Equation 5.

To complete Equation 40, we need the variance of 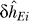 and *δh_Ii_*. It is convenient to break the former into two pieces, 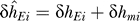 , where the first, *δh_Ei_*, is associated with the background neurons, and the second, *δh_mi_*, is associated with the foreground neurons (both will be defined shortly). Then, examining Equations 11–15, and performing a small amount of algebra, we find that

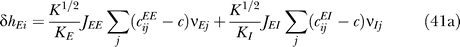


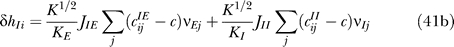
and

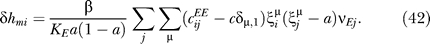
Here *δ_μ_*_,*v*_ is the Kronecker delta; it is 1 if *μ* = *ν* and zero otherwise. In addition, for notational convenience, we have returned the superscript “1” to *ξ_i_*. For the rest of the section, we will use 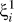 and *ξ_i_* interchangeably.

Let us focus first on the contribution from the background, Equation 41. Since 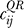 is equal to *c* on average, the mean of both terms on the right hand side of Equation 41 is zero. Moreover, these terms are uncorrelated, so their variances add. The variance of the *QR*th term is then

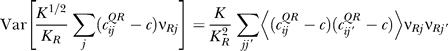
where the angle brackets represent an average over the distribution of 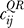 . Because 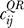 and 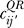 are independent when *j* ≠ *j′*, only terms with *j* ≠ *j′* produce a nonzero average. Thus, all we need is the variance of 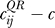 , which is given by

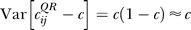
(the last approximation is valid because, as mentioned above, we are assuming *c* ≪ 1). Performing the sums over *j* and *j′* and collecting terms, we have

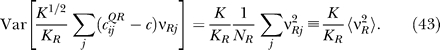
The term on the right-hand side, 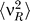 , is the second moment of the firing rate of the neurons in pool *R*. Inserting Equation 43 into 41, we find that

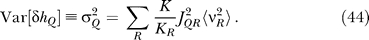



The last quantity we need is the variance of *δh_m_*. A naive approach to computing it proceeds along lines similar to those described above: assume all the terms in the sum over *j* and *μ* in Equation 42 are independent, so that the variance of *δh_m_* is just *pN_E_* (the number of terms in the sum) times the variance of each term. This yields, with rather loose notation for averages and ignoring the 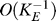 correction associated with *μ* = 1,

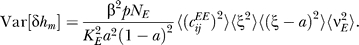
All the averages in this expression are straightforward: 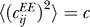 , 〈*ξ* ^2^〉 = *a*, 〈(*ξ* − *a*)^2^〉 = *a*(1 − *a*), and 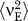 was defined in Equation 43. Putting all this together and defining *ρ* ^2^ to be the variance of *δh_m_*, we have

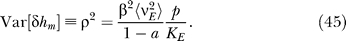



While Equation 45 turns out to be correct, our derivation left out a potentially important effect: correlations between the patterns, 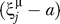 , and the firing rates, ν*_Ej_* in Equation 42. These correlations, which arise from the recurrent feedback, turn out to scale as *c*, and so can be neglected [32,35,70,71]. Rather than show this here, we delay it until the end of the section (see the section “Loop corrections vanish in the small *c* limit”).

To write our mean-field equations in a compact form, it is convenient to define the total excitatory variance,

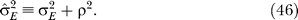
Then, combining Equations 3, 40, 44, and 45, the mean-field equations become

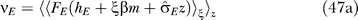


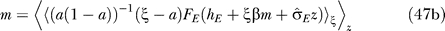


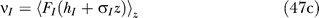


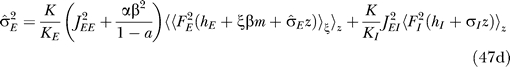


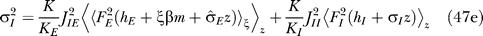
where the subscript *z* indicates a Gaussian average,

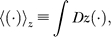
and, recall, *α* = *p*/*K_E_* (Equation 28).

Finally, it is convenient to explicitly perform the averages over *ξ* that appear in Equation 47. Defining

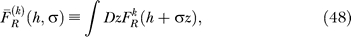
the relevant averages become

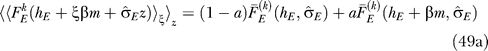


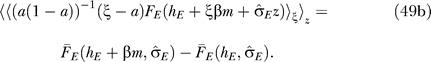
The functions 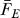 and 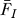 that we used in Equation 17 are equivalent to the ones defined in Equation 48, although we had suppressed the dependence on the standard deviation and dropped the superscript.

Equation 47 constitutes our full set of mean-field equations. A key component of these equations is that the number of memories, *p*, enters only through the variable *α*, which is *p*/*K_E_*. Thus, the number of memories that can be embedded in a network of this type is linear in the number of connections.

### Loop corrections vanish in the small *c* limit.

To correctly treat the loop corrections in our derivation of the variance of *δh_m_*, we need to be explicit about the correlations between the patterns, 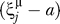 , and the firing rates, ν*_Ej_*, in Equation 42. We start by defining the *i*-dependent overlap, 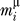 , as

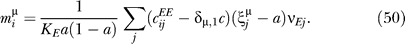
Inserting this into Equation 42 leads to

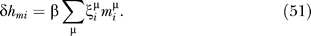
Each of the terms 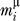 is a Gaussian random variable whose variance must be determined self-consistently. This can be done by inserting Equation 3 into Equation 50 to derive a set of nonlinear equations for the 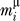 . There are two types of terms to consider: the activated memory, for which *μ* = 1, and the non-activated memories, for which *μ* ≠ 1. However, in the large *p* limit we can safely ignore the one term corresponding to *μ* = 1. Thus, considering the contributions from memories with *μ* ≠ 1, we have

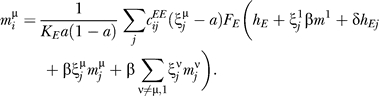
Taylor expanding around 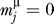 and defining

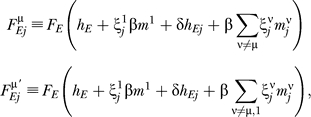
where a prime denotes a derivative, we have

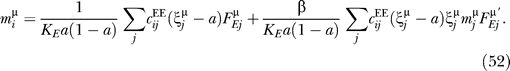
We can write Equation 52 in matrix form as

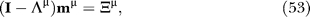
where **I** is the identity matrix, the *i*th component of **m** *^μ^* is equal to 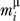 , and the matrices Λ*^μ^* and Ξ*^μ^* are given by

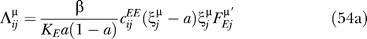


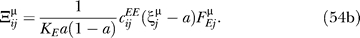



To solve Equation 53, we need to invert **I** − Λ, in general a hard problem. However, what we show now is that Λ has only one *O*(1) eigenvalue, with the rest 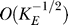 . This allows us to write the inverse in terms of a single eigenvector and adjoint eigenvector, a simplification that allows us to perform the inversion explicitly.

The spectrum of the random matrix, Λ*^μ^*, is determined primarily by the mean and variance of its components [72]. In the large *N_E_* limit, these are given by

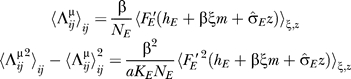
where 〈···〉*_ij_* indicates an average over *i* and *j*, and we used the fact that 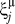 and 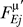 are independent.

Given that Λ*^μ^* is an *N_E_* × *N_E_* matrix, the fact that the mean and variance of its elements are 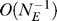 and *O*((*K_E_N_E_* )^−1^), respectively, implies that it has one eigenvalue that is *O*(1) and *N_E_* − 1 eigenvalues that are 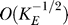 [72]. Letting **v** *_k_* and 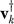 be the eigenvector and adjoint eigenvector of Λ*^μ^* whose eigenvalue is *λ_k_*, we can solve Equation 53 for **m** *^μ^*,

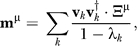
where “·” represents dot product. Letting *k* = 0 correspond to the *O*(1) eigenvalue and explicitly separating out this component, the expression for **m** *^μ^* becomes

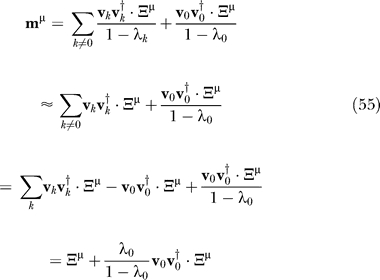
and

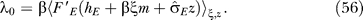
Since **v**_0_ and 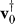 are vectors whose components are all the same, without loss of generality we can choose **v**_0_ = (1,1,…1)/*N_E_* and 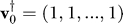 . Combining this choice with Equation 55 and using Equation 54b for Ξ*^μ^*, we have

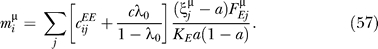



We are now in a position to return to Equation 51 and compute the variance of *δh_m_* (which, recall, is denoted *ρ* ^2^). Treating, as usual, all the terms in Equation 51 as independent, we have

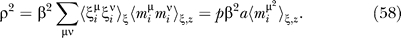
To compute 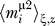 we use Equation 57 and the fact that the off-diagonal elements average to zero, and we find that

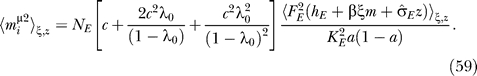
To derive this expression, we again used 〈(*ξ* − *a*)^2^〉 = *a*(1 − *a*).

Our final step is to insert Equation 59 into Equation 58. Ignoring the two terms in brackets in Equation 59 that are a factor of *c* smaller than the first, and using the fact that 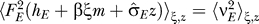 , this leads to the expression for *ρ* ^2^ given in Equation 45. Consequently, loop corrections vanish, and we can use our naive estimate for the variance of *δh_m_*.

Ignoring the two terms in brackets in Equation 59 is strictly correct for infinitely diluted networks; i.e., networks with *c* → 0. When *c* is nonzero but small, the terms in the brackets can be ignored safely unless λ_0_ → 1. However, as we now show, λ_0_ → 1 is precisely the point where the background becomes unstable. Thus, it is not a regime in which we can operate.

The significance of the limit λ_0_ → 1 can be seen by replacing Equation 53 by its dynamical counterpart (see Equation 1),

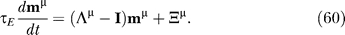



When the largest eigenvalue of Λ*^μ^* exceeds 1, the unactivated memories become unstable, and retrieval of just one memory is impossible. As discussed above, the largest eigenvalue of Λ*^μ^* is *λ*_0_. Consequently, loop corrections are necessarily important (no matter how dilute the network is) at precisely the point where the unactivated memories, and thus the background, become unstable.

### Stability analysis.

To determine stability, we need to write down time-evolution equations for the order parameters, and then linearize those around their fixed points. For *ν_E_*, *ν_I_*, and *m*, which are linear combinations of the firing rates, this is straightforward—we simply insert their definitions, Equation 16, into the time-evolution equations for the individual firing rates, Equation 1. For the variances, 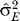 and 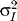 , the situation is much more difficult, as these quantities do not admit simple time-evolution equations [73]. Fortunately, we expect the effects of the variances to be small—as discussed in the main text, their primary effect is to smooth slightly the gain functions, something that typically (although presumably not always) stabilizes the dynamics. Alternatively, if we assume that the variances are functions of *ν_E_*, *ν_I_*, and *m*, (meaning we give them instantaneous dynamics), we can rigorously neglect them. This is because derivatives of the gain functions with respect to *ν_E_* and *ν_I_* are large, on the order of *K* ^1/2^, while derivatives with respect to the variances are *O*(1). Thus, as a first approximation, we will ignore these variables, and consider only the dynamics of *ν_E_*, *ν_I_*, and *m*. Because of this approximation, we expect our stability boundaries to be off by a small amount.

Combining Equation 1 and Equation 47, the time-evolution equations for *ν*_E_, *ν*_I_, and *m* may be written

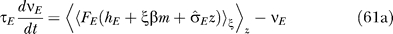


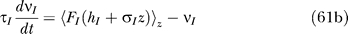


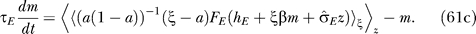
To simplify notation, it is convenient to define

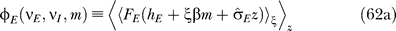


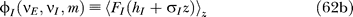


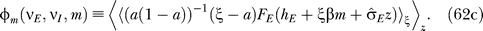
Then, linearizing Equation 61 by letting *ν_E_* → *ν_E_* + *δν_E_*, *ν_I_* → *ν_I_* + *δν_I_*, and *m* → *m* + *δm*, we have

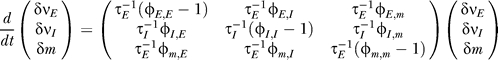
where the notation *φ_a,b_* indicates a derivative of *φ_a_* with respect to the argument specified by *b* (for example, *φ_E,I_* = ∂*φ_E_*/∂ν*_I_* and *φ_I,M_* = ∂*φ_I_*/∂*m*). Since *φ_I_* is independent of *m* (which means *φ_I,m_* = 0), the equation for the eigenvalues, denoted *λ*, becomes

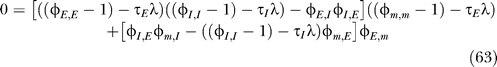



Equation 63 is a cubic equation in *λ*, and thus not straightforward to solve. However, in the large *K* limit it simplifies considerably. That's because derivatives with respect to *ν_E_* and *ν_I_* are *O*(*K* ^1/2^), which follows because the *φ*'s depend on *ν_E_* and *ν_I_* through *h_E_* and *h_I_*, and the latter are proportional to *K* ^1/2^ (see Equation 13). Defining the *O*(1) quantities

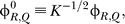

*R* = ν*_E_*,*ν_I_*,*m* and *Q* = *ν_E_*,*ν_I_*, Equation 63 becomes (ignoring *O*(*K* ^−1/2^) corrections)

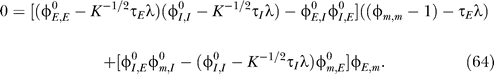



Examining Equation 64, it follows that if the eigenvalue, *λ*, is *O*(*K* ^1/2^), then the term *φ_m_*_,*m*_ − 1 and the last term in brackets can be neglected. There are two such eigenvalues, and they are given by

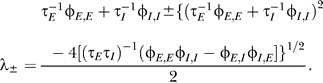
Both eigenvalues are negative if

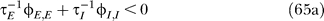


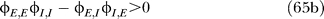
Since *φ_I_*_,*I*_ < 0, the first condition is satisfied if *τ_I_* is sufficiently small. For the second condition, from Equations 13, 20, and 62, we see that

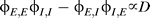
where the constant of proportionality is positive. Since the condition for the stability of the background is *D* > 0 [54], we see that 65b is satisfied whenever the background is stable. Thus, for *τ_I_* sufficiently small and the background stable, the two *O*(*K* ^1/2^) eigenvalues are negative.

The third eigenvalue is *O*(1), so when computing it we can drop all the *K* ^−1/2^ *λ* terms. Denoting this eigenvalue *λ_m_*, we thus have

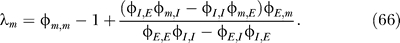
Using a prime to denote a derivative with respect to *h_E_* and noting that (see Equation 62)

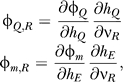

Equation 66 reduces to

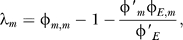
where prime denotes a derivative.

Comparing Equations 62 and 49, we see that *φ_E_*_,*m*_ = *aφ_m_*_,*m*_, which leads to

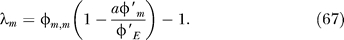
This expression strongly emphasizes the role of the coding level, *a*: if it were zero, the only stable equilibria would be those with *φ_m_*_,*m*_ < 1, which would imply high firing rates for foreground neurons (see Figure 1B).

Although Equation 67 tells us the stability of an equilibrium, it is not in an especially convenient form, as it does not allow us to look at a set of nullclines and determine instantly which equilibria are stable and which are not. However, it turns out that it is rather easy to determine the sign of *λ_m_* for a given set of nullclines simply by looking at them. To see how, we make use of the expressions for *φ_E_* and *φ_m_* (Equations 62a and 62c) to reduce the right-hand side of Equation 67 to an expression with a single derivative. Our starting point is the definition

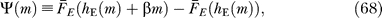
where *h_E_*(*m*) is given by Equation 26; the solutions of the equation Ψ(*m*) = *m* correspond to network equilibria. The advantage of this one-dimensional formulation is that, as we show below, the condition *λ_m_* < 0 is equivalent to *d*Ψ/*dm* < 1. Thus, by plotting the function Ψ(*m*) versus *m* and looking at its intersections with the 45° line, we can find the equilibrium values of *m*, and, more importantly, we can easily determine which of them is stable and which is unstable.

**Figure 10:**
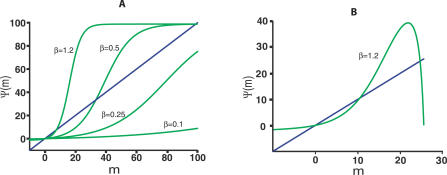
Stable Equilibrium Values of *m* Are Those with *d*Ψ/*dm* < 1 (A) Ψ(*m*) versus *m* for *a* = 0.001 and *β* = 0.1, 0.25, 0.5, and 1.2. The equilibrium values of *m* are the intersections of the curves Ψ(*m*) with the diagonal line. For small *β*, *m* = 0 is the only solution that is stable. For intermediate *β*, there are two additional non-zero solutions, one stable and one unstable. (B) Ψ(*m*) versus *m* for *a* = 0.05 and *β* = 1.2; the upper intersection is now at a biologically realistic firing rate. Note different scales compared to (A). Other parameters, as in Figure 2, are *J_EE_* = *J_IE_* = 1, J*_EI_* = −1.9, *J_II_* = −1.5, *h_Eex_* = 3, *h_Iex_* = 2.1.

To show that *d*Ψ/*dm* < 1 is equivalent to the condition *λ_m_* < 0, we note first of all that

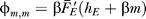


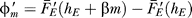


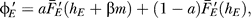
where, recall, a prime denotes a derivative. By combining these expressions with Equation 67, and performing a small amount of algebra, the condition *λ_m_* < 0 can be written

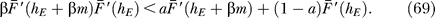
To see how this compares to *d*Ψ/*dm*, we use Equation 68 to write

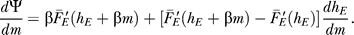
Then, using Equation 25, which tells us that

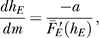
this expression becomes

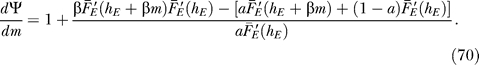
Comparing Equations 69 and 70, we see that the condition *d*Ψ/*dm* < 1 is equivalent to *λ_m_* < 0. Thus, it is only when Ψ(*m*) intersects the 45° line from above that, the equilibrium is stable. Since Ψ(*m*) is bounded, if there are three equilibria, the smallest one must be stable, the middle one unstable, and the largest one again stable. Thus, we can look at the nullcline plots and immediately determine stability (see below and Figure 10).

As an example, we revisit Figure 2. In terms of our specific form for the gain functions, Equation 23, and with *h*_E_(*m*) given by Equation 26, the equation for *m* becomes

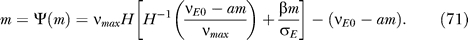
This equation is solved graphically in Figure 10A where we plot Ψ(*m*) versus *m* for the same values of *β* used in Figure 2 and with *a* = 0.005. Intersections with the 45° line correspond to solutions of Equation 71, and thus to network equilibria.

As we saw in the sections “Reduced mean-field equations in the infinite *K* limit” and “An example: Nullclines for a simple gain function,” the main factor that determines the number and location of the intersections, and thus the ability of the network to exhibit retrieval states, is *β*. For *β* = 0.1 and 0.25, there is just one intersection at *m* = 0, while for intermediate values of *β*, *β* = 0.5 and 1.2, two additional intersections appear. Increasing *β* even further moves one of the solutions to negative *m* and destabilizes the background, but this is not shown. We can now easily see that the curves in Figure 10A with *β* = 0.1 and 0.25 have a single stable intersection at *m* = 0 (meaning that the solutions with *m* = 0 in Figures 2A and 2B are stable); the curves with *β* = 0.5 and *β* = 1.2 have two stable intersections, one at *m* = 0 and one at large *m* (and thus the solutions at *m* = 0 in Figure 2C are stable, those at intermediate *m* are unstable, and those with large *m* are again stable).

Although we see bistability, the firing rate for the retrieval state is unrealistically high—on the order of 100 Hz, near saturation. As discussed in the main text, we can reduce the firing rate by increasing *a*. This is done in Figure 10B, where we plot Ψ(*m*) versus *m* but this time for *a* = 0.05 and *β* = 1.2. Again there are three intersections (corresponding to the three intersections between the *m*-nullcline with *β* = 1.2 and the *h_E_*-nullcline with *a* = 0.05 in Figure 2C). With this higher value of *a*, the upper intersection is now in a biologically realistic range.

### Retrieval states in the finite connectivity regime.

When we performed network simulations, we found that the memory strength, 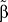 , did not exhibit exactly the predicted 1/*K* scaling. Here we ask whether the departure from predictions that we observed can be explained by finite *K* corrections. These corrections, as we will see shortly, are on the order of *K* ^−1/2^. Since in our simulations *K* is as small as 1,500, these corrections are potentially large.

Our starting point is the exact set of reduced mean-field equations, which is found by combining Equations 18 and 19,

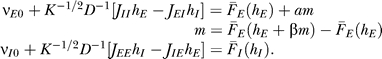
When *K* is large we can solve these equations by perturbing around the *K* → ∞ solutions, which we denote *h_E_*_0_, *m*_0_, and *h_I_*_0_ (these are the solutions to Equation 21). The zeroth step in this perturbation analysis is to replace *h_E_* and *h_I_* by *h_E_*_0_ and *h_I_*_0_ where they appear in brackets (and thus multiply *K* ^−1/2^). This gives us a new set of equations,

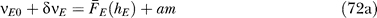


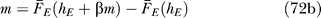


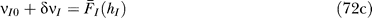
where

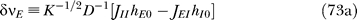


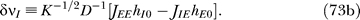



For the inhibitory firing rate, it is easy to see the effect of finite *K*: *h_I_* is shifted relative to *h_I0_* by an amount proportional to *δν_I_*. Only slightly more difficult are *h_E_* and *m*, for which we have to consider how *δν_E_* affects the nullclines. Fortunately, only the *h_E_*-nullcline is affected, and we see that it shifts in a direction given by the sign of *δν_E_*. In particular,

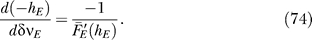
(We consider −*h_E_* since, by convention, we plot our nullclines in a space with −*h_E_* on the *y*-axis.) Thus, if *δν_E_* is positive then the *h_E_*-nullcline shifts down relative to *h_E0_*, while if it is negative the nullcline shifts up.

In our simulations we set *β* to *β_min_*, the minimum value of *β* that allows retrieval of one memory. To determine how *K* affects *β_min_*, then, we need to know how to adjust *β* so that we keep the grazing intersection as *K* changes. Fortunately, the *h_E_*-nullcline depends on *K* but not on *β*, and the *m*-nullcline depends on *β* but not on *K*. Thus, all we need to know is how the *m*-nullcline changes with *β*. Using Equation 72b, it is easy to show that at fixed *m*,

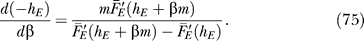
The numerator in this expression is clearly positive, and, for equilibria to the left of the peak of the *m*-nullcline, the denominator is also positive (see the section “Stability analysis”). Thus, increasing *β* causes the *m*-nullcline to move up.

**Figure 11:**
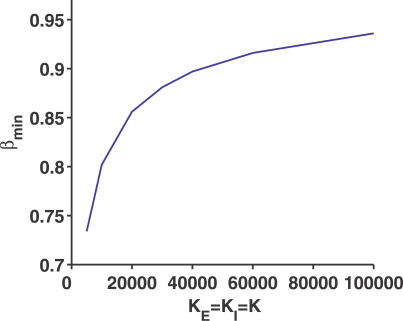
The Effect of Finite *K* Corrections on *β_min_* The minimum value of *β* at which a single stored pattern can be retrieved successfully, *β_min_*, decreases as *K* decreases. The parameters are the same as Figure 2 with *a* = 0.05.

Combining Equation 74 and 75, we have the following picture,

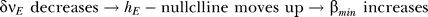
where “up” corresponds to movement in the *m* −(−*h_E_*) plane. To complete the picture, we need to know how *δν_E_* depends on *K*. From 73, we see that *δν*_*E*_ ∝ *K* ^−1/2^ [*J_II_h_E_*_0_ − *J_EI_h_I_*_0_] = *K* ^−1/2^[−|*J_II_*| *h_E_*_0_ + |*J_EI_*|*h_I_*_0_]. Thus, whether *δν_E_* is an increasing or decreasing function of *K* depends on whether |*J_II_*|*h_E_*_0_ is larger or smaller than |*J_EI_*|*h_I0_*. However, as we have seen, typically *h_E_* is negative. Thus, we expect *δν_E_* to be proportional to *K* ^−1/2^ with a positive constant of proportionality, which means that *δν_E_* is a decreasing function of *K*. Combining that with the above picture, we conclude that when *K* increases, *β_min_* also increases. This is shown explicitly in Figure 11. Moreover, it was exactly what we saw in our simulations: *β_min_* (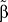 in Table 1) was larger than predicted when we increased *K* (compare 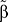 with 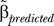 in Table 1).

### Fine-tuning in the learning rule.

In the model described here, the structured part of the synaptic weights scale as *K* ^−1^, whereas the background scales as *K* ^−1/2^. This appears to require fine-tuning, since adjustments to the weights during learning of the attractors have to be a factor of *K* ^1/2^ times smaller than the background weights; a factor that can be as high as 100.

The first question to ask, then, is: exactly how big is the fine-tuning problem? In other words, how much noise can we add to the learning rule without having a huge effect on the storage capacity? This can be answered by considering a learning rule in which the weight changes during learning a pattern are not quite perfect. Specifically, let us consider the following modification of Equation 4,

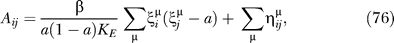
where the 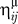 are zero-mean, uncorrelated random variables with variance 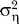 . The additional noise in this learning rule increases the variance of the quenched noise by an amount 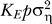 . As a result, if

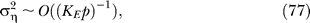
the effect on storage capacity is an *O*(1) increase in the quenched noise, and thus the storage capacity still scales as *K_E_*.

With the scaling in Equation 77, weight changes during learning of each pattern is a factor of *p* ^1/2^ smaller than the background weights, and therefore the amount of fine-tuning depends on how many patterns are stored. Because of the low storage capacity found in these networks (at most 2.5% [23]), even when *K* is as large as 10,000, *p* ^−1/2^ is on the order of 6%.

We should also point out that it is possible for the weight changes associated with the structured part of the connectivity to be on the same order as the background, although at the expense of storage capacity. Let us consider a third learning rule in which each synapse has a probability *q* of changing its value during learning,

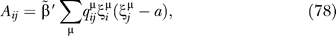
where the 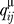 are Bernoulli variables; 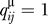 with probability *q* and 0 with probability 1 − *q*. Let us define the coupling strength slightly differently than in Equation 10,

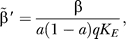
where, as usual, *β* ∼ *O*(1). With this definition, the mean memory strength, 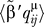 , is again *β/K_E_a*(1 − *a*), as in Equation 10. But by setting 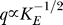 , the synaptic weight change—if there is one—is 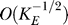 , just as it is for the background weights. However, there is a major drawback: as is easy to show, the variance associated with the structured part of the connectivity increases by a factor of *K_E_*, so the maximum number of patterns scales as 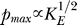 rather than *K_E_*. We thus use Equation 4 for *A_ij_* in all of our analysis.

## Abbreviations

CV: coefficient of variation
PSP: postsynaptic potential
